# Laryngeal evidence for the first and second *passaggio* in professionally trained sopranos

**DOI:** 10.1371/journal.pone.0175865

**Published:** 2017-05-03

**Authors:** Matthias Echternach, Fabian Burk, Marie Köberlein, Andreas Selamtzis, Michael Döllinger, Michael Burdumy, Bernhard Richter, Christian Thomas Herbst

**Affiliations:** 1 Institute of Musicians’ Medicine, University of Freiburg Medical Center, Faculty of Medicine, University of Freiburg, Freiburg, Germany; 2 Royal Technical University, Music Acoustics. Lindstedtsvägen 24, Stockholm, Sweden; 3 Division of Phoniatrics and Pediatric Audiology at the Department of Otorhinolaryngology Head & Neck Surgery, University Hospital Erlangen, Medical School, Waldstrasse 1, Erlangen, Germany; 4 Department of Medical Physics, University of Freiburg Medical Center, Faculty of Medicine, University of Freiburg, Freiburg, Germany; 5 Laboratory of Bio-Acoustics, Department of Cognitive Biology, University of Vienna, Althanstraße 14, Vienna, Austria; Northwestern University, UNITED STATES

## Abstract

**Introduction:**

Due to a lack of empirical data, the current understanding of the laryngeal mechanics in the *passaggio* regions (i.e., the fundamental frequency ranges where vocal registration events usually occur) of the female singing voice is still limited.

**Material and methods:**

In this study the first and second *passaggio* regions of 10 professionally trained female classical soprano singers were analyzed. The sopranos performed pitch glides from A3 (*ƒ*_o_ = 220 Hz) to A4 (*ƒ*_o_ = 440 Hz) and from A4 (*ƒ*_o_ = 440 Hz) to A5 (*ƒ*_o_ = 880 Hz) on the vowel [iː]. Vocal fold vibration was assessed with trans-nasal high speed videoendoscopy at 20,000 fps, complemented by simultaneous electroglottographic (EGG) and acoustic recordings. Register breaks were perceptually rated by 12 voice experts. Voice stability was documented with the EGG-based sample entropy. Glottal opening and closing patterns during the *passaggi* were analyzed, supplemented with open quotient data extracted from the glottal area waveform.

**Results:**

In both the first and the second *passaggio*, variations of vocal fold vibration patterns were found. Four distinct patterns emerged: smooth transitions with either increasing or decreasing durations of glottal closure, abrupt register transitions, and intermediate loss of vocal fold contact. Audible register transitions (in both the first and second *passaggi*) generally coincided with higher sample entropy values and higher open quotient variance through the respective *passaggi*.

**Conclusions:**

Noteworthy vocal fold oscillatory registration events occur in both the first and the second *passaggio* even in professional sopranos. The respective transitions are hypothesized to be caused by either (a) a change of laryngeal biomechanical properties; or by (b) vocal tract resonance effects, constituting level 2 source-filter interactions.

## Introduction

The frequency range of the singing voice is not a seamless continuous domain. Instead, at certain fundamental frequencies the voice quality may change abruptly. Vocal frequency regions with similar sound characteristics are commonly referred to as vocal registers [1] and abrupt shifts from one register to another are frequently called register breaks. Although vocal registers have been empirically described and physiologically analyzed as early as 1835 [2], vocal registers are not yet fully understood. To date, there is still no complete consensus regarding the terminology of singing voice registers, particularly concerning their number and definition [1,3–15]. It is assumed that perceptive differences of registers could be related to differences in activities of laryngeal muscles [2,10,13,16], differences in vocal tract resonances [12,17–20], interactions of the subglottal resonances with the voice source [21], or interactions of vocal tract resonances with the voice source [22–25]. Register changes are frequently accompanied by acoustic variations [12,26–28] and fundamental frequency jumps [29,30]. In these cases, the sudden change of vocal fold oscillation patterns in untrained voices is often assumed to be a consequence of non-linear biomechanical properties of the vocal folds and/or interactions with the supra- or subglottal vocal tracts [22,24,31]. In contrast to untrained voices, western classically trained singers are largely able to avoid such perceptive register differences [32]. However, the mechanisms to prevent biomechanical instabilities are still not understood in detail.

In male voices, most attention is directed towards the transition from modal (or chest) register to the falsetto register. In particular, it was shown for male voices, that vocal fold oscillatory patterns might change in the fundamental frequency (*ƒ*_o_) range where registration events typically occur [2,13,33], often denoted as the *passaggio* region. In addition to changes of vocal fold vibratory patterns, articulatory adjustments play an important role in the *passaggio*. For example, while the vocal tract shape remains nearly stable as singers advance from the modal register to the unmodified falsetto register, professional male singers introduce considerable changes into their vocal tract geometry when transitioning into their upper stage voice registers (stage falsetto for professional male altos and stage voice above the passaggio for tenors, respectively) [34–37]. This suggests that vocal tract changes could be used as a stabilizing factor for professional singing across the male *passaggio*.

The vocal registers of female voices are less well understood. Some authors suggest that female singers not only have (apart from the fry register) a first *passaggio* (also called *primo passagio*) from modal or chest register to a middle or head register, but also a second *passaggio* (*secondo passaggio*) from middle or head register to an upper register [6,12,14,38–41]. Unfortunately, the terminology used to describe these registers is inconsistent and poorly defined. Furthermore, there might be subdivisions within registers. Based on electromyographic data, Kochis-Jennings et al. [10] propose that thyroarytenoid muscle activity might differ among what the authors denote as “chest”, “chestmix”, “headmix”, and “head” register. Herbst et al. found that the degree of adduction of both the cartilaginous and membranous portions of the vocal folds could be controlled independently, and that such control could lead to production of different timbres within different registers [42]. As a consequence, the degree of adduction of the vocal process might also contribute to the differences observed in female voices [10]. Some singers also have been seen to produce a whistle register above 1000 Hz, suggesting the presence of a third *passaggio* [38,39,43,44].

The female first *passaggio* is often assumed to be caused by a change in vocal fold oscillation patterns [6,29,39,45]. It has been shown that both the transglottal flow pulse and the electroglottographic (EGG) signal changes through the first *passaggio*, resulting in increased open quotients [29,45]. Furthermore, radiologic studies have revealed that the distance between the arytenoid and the thyroid cartilages changes within the first *passaggio* [41]. Also, the sound spectrum has been found to differ between the modal and middle register, exhibiting a stronger first harmonic in the middle register, as compared to the second harmonic [46]. The *ƒ*_o_ region where this register shift occurs is located only slightly above the region of the respective register shift from modal to falsetto register in males [6,12,40,41]. Although there is some evidence for changes of vocal fold oscillation patterns in this *passaggio* [6,47], there are only a few studies analysing vocal fold vibration. In 1960, Rubin and Hirt [48] found vocal fold oscillatory differences between what the authors denoted as *chest* and *falsetto* for their female singers. Svec et al. [39] analysed this *passaggio* in a single untrained subject and observed a decrease of arytenoid adduction for the middle or head register as compared to the modal register. Furthermore, it was found that the videokymographically derived closed quotient was decreasing from the middle to the head register [39].

In contrast to the first *passaggio*, empirical information on the second *passaggio* is still scarce and somewhat conflicting. While some authors suggest a resonatory phenomenon which occurs when *ƒ*_o_ reaches the first vocal tract resonance [38], other studies propose a vibratory phenomenon, suggesting that it is also possible that vocal fold oscillation patterns are altered in the second passaggio region [19,39].

Supporting the idea of resonatory phenomena, the *ƒ*_o_ region of the second *passaggio* often corresponds to the frequency region where vocal tract shape adjustments [40,49,50] and the resulting alterations of vocal tract resonances can often be observed (i.e., the so called “formant tuning” [43,51,52]).

With reference to laryngeal adjustments, Garnier et al. [19] found a decrease of EGG amplitude and an increase of EGG open quotient between the pitches of G4 and D6. These changes in vocal fold vibration were rather gradual and not necessarily accompanied by acoustic changes induced by resonatory phenomena [19]. This important evidence notwithstanding, there is a lack of empirical data describing vocal fold oscillation patterns in the second *passaggio*. Garnier et al. [19] discuss only one single high speed videoendoscopy (HSV) recording acquired using rigid laryngoscopy at a limited frame rate of 2,000 fps in a single “non-expert” subject. They observed only minor variations of vocal fold vibration during the second *passaggio*. In contrast, using videokymography (i.e., assessment of vocal fold vibration along only one single line perpendicular to the glottal axis at around 8,000 fps [53]) Svec et al. [39] found an *ƒ*_o_ jump in their single untrained subject study at an *ƒ*_o_ of 650 Hz. Unfortunately, these noteworthy pilot examinations were limited both technically and in their number of subjects. Furthermore, both studies were conducted using rigid endoscopy, a method that forces the participants to introduce considerable changes into the configuration of their vocal tracts while phonating [19,39], potentially influencing the participants’ habitual strategies throughout the *passaggi*.

Summarizing, the exact nature of the second *passaggio* is still not understood in detail. Furthermore, the vocal fold oscillation patterns through both *passaggi* have not been recorded nor analysed in detail, partly due to limitations of frame rates and spatial resolution in previous investigations utilizing endoscopy. It is thus the purpose of this study to analyse vocal fold oscillation patterns in a greater number of participants, using state of the art HSV equipment with a sufficiently high temporal and spatial resolution, employing flexible endoscopy in order to allow the habitual articulatory gestures of the participants. Because untrained singers might have problems in reaching higher pitches above the second *passaggio*, the study focuses on professional singers.

## Material and methods

### Participants and phonatory tasks

After approval from the Freiburg University Ethical Comittee (nr. 380/12), ten professional female singers, all of them sopranos trained in classical singing, were included in this study. All subjects gave their written consent to participate in this study. The classification of the participants according to the Bunch and Chapman taxonomy [54] is shown in Table 1. In all of the participants, laryngoscopic examination prior to data acquisition revealed no signs of vocal fold pathology.

**Table 1:** Demographic information and taxonomy according to Bunch and Chapman [54] for all participants.

Participant	Age	Years of Training and professional activity	Taxonomy	Description
S1	52	>30	3.1c	National/Big City–Opera Chorus
S2	30	5	7.1	Full-time Voice Student–Postgraduate specialist training courses
S3	45	>25	3.1a	National/Big City–Opera Major Principal
S4	25	2	7.1	Full-time Voice Student–Postgraduate specialist training courses
S5	37	15–20	3.1a	National/Big City–Opera Major Principal
S6	30	10	2.1	International–Opera principal
S7	26	7	7.1	Full-time Voice Student–Postgraduate specialist training courses
S8	24	4	7.1	Full-time Voice Student–Postgraduate specialist training courses
S9	25	6	7.1	Full-time Voice Student–Postgraduate specialist training courses
S10	32	10	4.1b	Regional/Touring–Minor Principal

The participants were asked to perform two upward pitch glides, one from pitch A3 (*ƒ*_o_ = 220 Hz) to A4 (*ƒ*_o_ = 440 Hz) and another from A4 (*ƒ*_o_ = 440 Hz) to A5 (*ƒ*_o_ = 880 Hz) on the vowel [iː]. The two pitch glides cover the *ƒ*_o_ regions where the first and second *passaggio*, respectively, are typically found [6,12,19,29,39,40,55]. The vowel [iː] was chosen in order to ensure best visibility of the vocal folds, additionally preventing major gag reflexes due to increased pharynx width. The participants were asked to sing both pitch glides using their professional “stage voice” at comfortable loudness, theoretically avoiding major voice quality differences. The glide was to be performed over a time period of approximately one second. The total number of acquired pitch glides was two per subject for ten subjects, a total of 20 glides.

### Data acquisition

The data acquisition setup is described in detail in a previous publication [56]: Laryngeal endoscopy was performed trans-nasally using an ENF GP endoscope (Olympus, Hamburg, Germany) with a 38mm C-mount adapter (Karl Storz, Tuttlingen, Germany) and a 300W light source (Storz, Tuttlingen, Germany). Endoscopic laryngoscopy was recorded with a Fastcam SA-X2 high-speed video camera (Photron, Tokyo, Japan) operated at a frame rate of 20,000 frames per second and a spatial resolution of 386 x 320 pixels. No anaesthetic medication was given for the trans-nasal endoscopic approach.

Simultaneous with the HSV recording, the acoustic and EGG signals were recorded with a IMK SC 4061 microphone (DPA microphones, Alleroed, Denmark) and an EG2-PCX2 electroglottograph (Glottal Enterprises, Syracuse, NY, USA) using a data acquisition board (National Instruments, Austin, USA). The simultaneous recording of both HSV data and acoustic and EGG signals was performed using the PFV Viewer Software (Version 3660, Photron, Tokyo, Japan). As both the HSV camera and the data acquisition board were operated at a sampling frequency of 20,000 Hz, the PFV software allowed for time-synchronized acquisition of all signals. The accuracy of the synchronization was tested by simultaneous playback of a test signal consisting of TTL pulses to the data acquisition board and a blinking LED signal (to be acquired by the camera). Using this method, the accuracy was determined to be one frame, which is equivalent 50 μs.

### Perceptual rating

It could be expected that, in contrast to untrained voices, the professional singers participating in this study were able to avoid great sound quality differences during the pitch glides. In order to evaluate if a register transition was perceptually noticeable, the acoustic signals of both pitch glides per participant were played back in randomized order to 12 experts for a perceptual rating. These expert raters were either professors of singing (n = 2) or full time singing students at a German University of music with a minimum professional voice training of 4 years (n = 10). The experts were asked to rate the acoustic recordings (n = 10 subjects x 2 phonatory tasks = 20 ratings) on a scale from 1 (no perceivable register event) to 5 (maximum perceivable register event). For all raters, the rating was performed in the same room with the same headphones and the same loudness. In order to estimate the reliability of the ratings, the stimuli of one subject were provided twice in the set, and the Intra-class Correlation Coefficient (ICC [57]) of the raters was calculated. The averaged measured ICC was 0.85, indicating a good degree of rating consistency.

### Data processing

All high-speed videos were subjected to three pre-processing steps, as described previously [56]: the honeycomb structure introduced by the optics of the flexible endoscope was removed using a frequency-selective filter in the Fourier domain; the acquired images were rotated to represent the glottal midline exactly vertically with respect to the image frame; last, the video was cropped to a region of interest containing the vibrating vocal folds. Then, glottal segmentation, i.e., semi-automatic extraction of the time-varying medio-lateral deflections of the vocal folds from the video footage, was performed using the Glottis Analysis Tools software (Denis Dubrovskiy and Michael Döllinger, FAU Erlangen-Nürnberg, Germany) [58]. The time-varying area of the glottis (i.e., the air space between the vibrating vocal folds, as seen from the top) was computed based on the glottal segmentation data, resulting in the glottal area waveform (GAW).

The electroglottographic (EGG) signal is proportional to changes of the relative vocal fold contact area during vocal fold vibration [59]. It is thus well suited for documenting the vocal fold oscillatory effects of any potentially occurring register transitions or instabilities during the examined pitch glides. This was achieved by calculating the sample entropy of the cycle-separated EGG signal [60,61]. Sample entropy is defined [60,61] as “the negative natural logarithm of the conditional probability that two sequences similar for m points remain similar at the next point, where self-matches are not included in calculating the probability.” The sample entropy was chosen over other irregularity measures because it is not sensitive to changes of *ƒ*_o_ when calculated using the cycle-separated (rather than the raw) EGG signal. This was verified by analysing synthesized stereotypical EGG signals representing the tasks of this study. These synthesized EGG signals had durations of one second, and the *ƒ*_o_ was changed from 220 Hz to 440 Hz and from 440 Hz to 880 Hz, respectively, within an interval of 50 ms centred around a time offset of 0.5 s. There was no effect of the *ƒ*_o_ variation on the sample entropy. In this study, the cycle based sample entropy was calculated based on the time series of the first two Discrete Fourier Transform components, termed “Fourier Descriptors”, of the analyzed EGG signals (FDSE_c_). The respective calculations were performed with the algorithm developed by Selamtzis and Ternström [60] which is described in detail in the supplementary material S1 Text.

In order to align all phonations of all participants at a temporal instant for each pitch glide signifying the moment of maximum change in the vocal fold vibration pattern, all analyzed EGG signals were divided into consecutive sequences of 25 ms, and the mean FD-based sample entropy was calculated for each of these segments. For the remainder of this manuscript, the term “window based FD sample entropy” (FDSE_w_) is used to denote this parameter. The EGG signals were chosen over the GAW signals since they had a better signal-to-noise ratio and did not suffer from potential effects of endoscope movement or rotation, thus being less susceptible to spurious sample entropy results. The segment with the maximum FDSE_w_ value within each EGG signal for each pitch glide was determined. Centred at this segment, a total of 11 segments (denoted as windows -5 to window 5 –see below and figures), each having a duration of 25 ms, were considered for further analysis. For each of these analysis windows, the following parameters were computed: FDSE_w_, open quotient of the GAW signal (OQ_GAW_, i.e., the relative, time-normalized duration of glottal opening per vocal fold vibratory cycle) and “between-window” variation thereof, and respective glottal opening and closing patterns.

Glottal opening and closing patterns were determined with a novel custom algorithm devised and implemented in Python by author C.T.H. For each data point along the anterior-posterior glottal axis, the time-varying medio-lateral vocal fold displacement was assessed. The instants where the respective vocal fold trajectory diverged from and converged to zero, respectively (indicating beginning and termination of glottal opening at a certain offset along the anterior-posterior (A-P) glottal midline), were divided by the respective duration of each glottal cycle within each analysis window (-5 to 5) for each pitch glide. The resulting cycle-by-cycle data for each analysis window were averaged and plotted as a function of medio-lateral vocal fold displacement along the A-P axis and normalized intra-cycle time. The resulting graphs show the spatio-temporal opening and closing patterns of the vocal folds for all the eleven analysis windows. For each of these analysis windows, two contours leading from the anterior to the posterior ends of the vocal folds were plotted: The left contour (occurring earlier within the normalized intra-cycle temporal dimension) shows the pattern for glottal opening and the right contour that for glottal closing.

## Results

Both pitch glides could be performed by all participants. Due to an equipment malfunction, the HSV data for the lower pitch glide of participant S6 had to be excluded from the analysis. Overall, the vibrating vocal folds were well visible in HSV for all recordings, allowing the segmentation of the glottis for all glides. The only exception was the upper pitch glide of participant S6, in which the visibility of the vocal folds was obstructed by a retracted epiglottis during the last portion of the recording.

**Fig 1:**
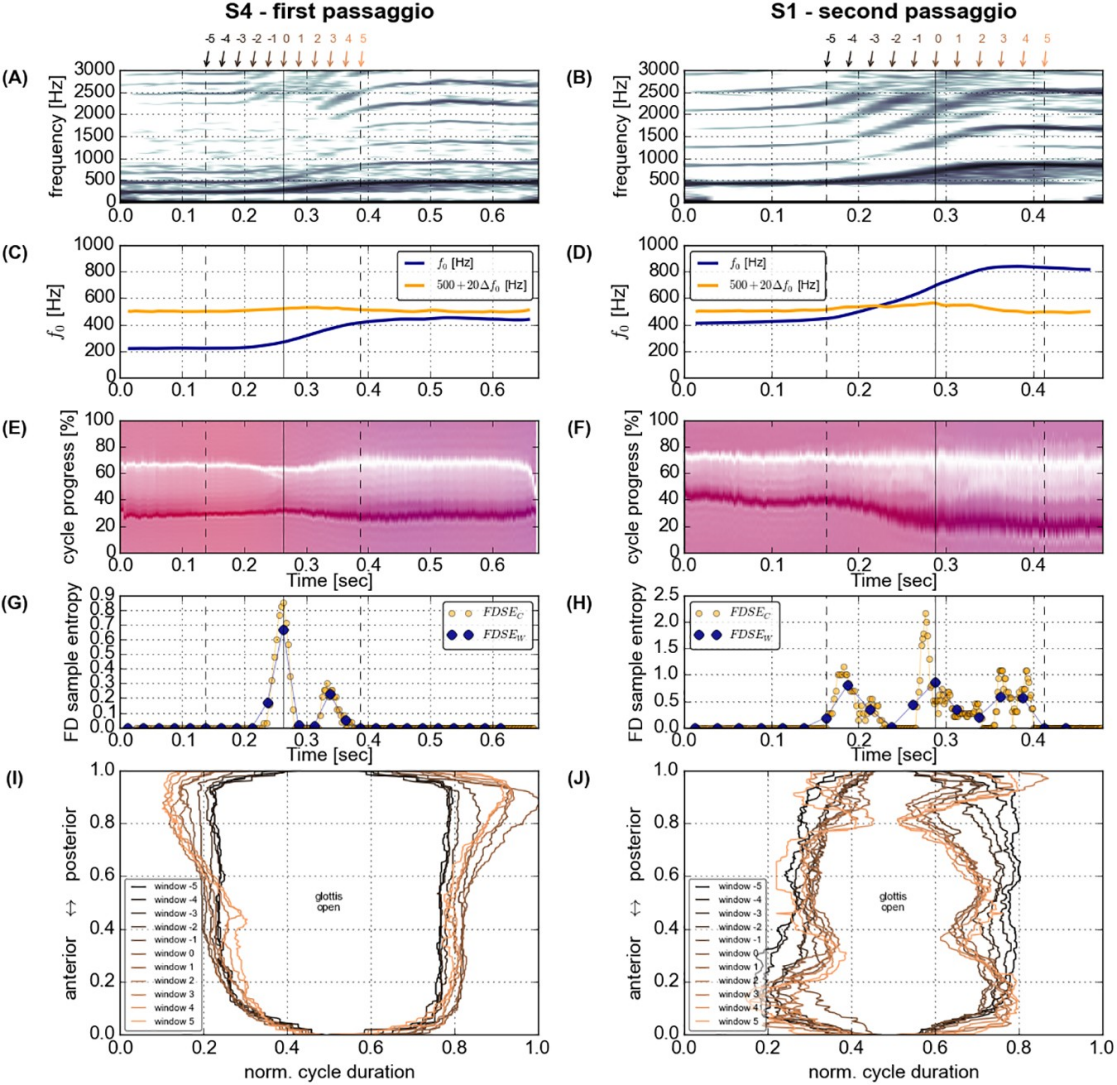
Acoustic spectrogram (window length 1024 frames, 65 dB dynamic range, A and B), time-varying fundamental frequency (*ƒ*_o_ and its first derivative *Δƒ*_o_, C and D), dEGG Wavegram (E and F), cycle based (c) and windows based (w) Fourier Descriptors Sample Entropy (FDSE) (G and H), and summary of glottal opening and closure patterns (I and J) for participants S4 (first *passaggio*) and S1 (second *passaggio*).

**Fig 2:**
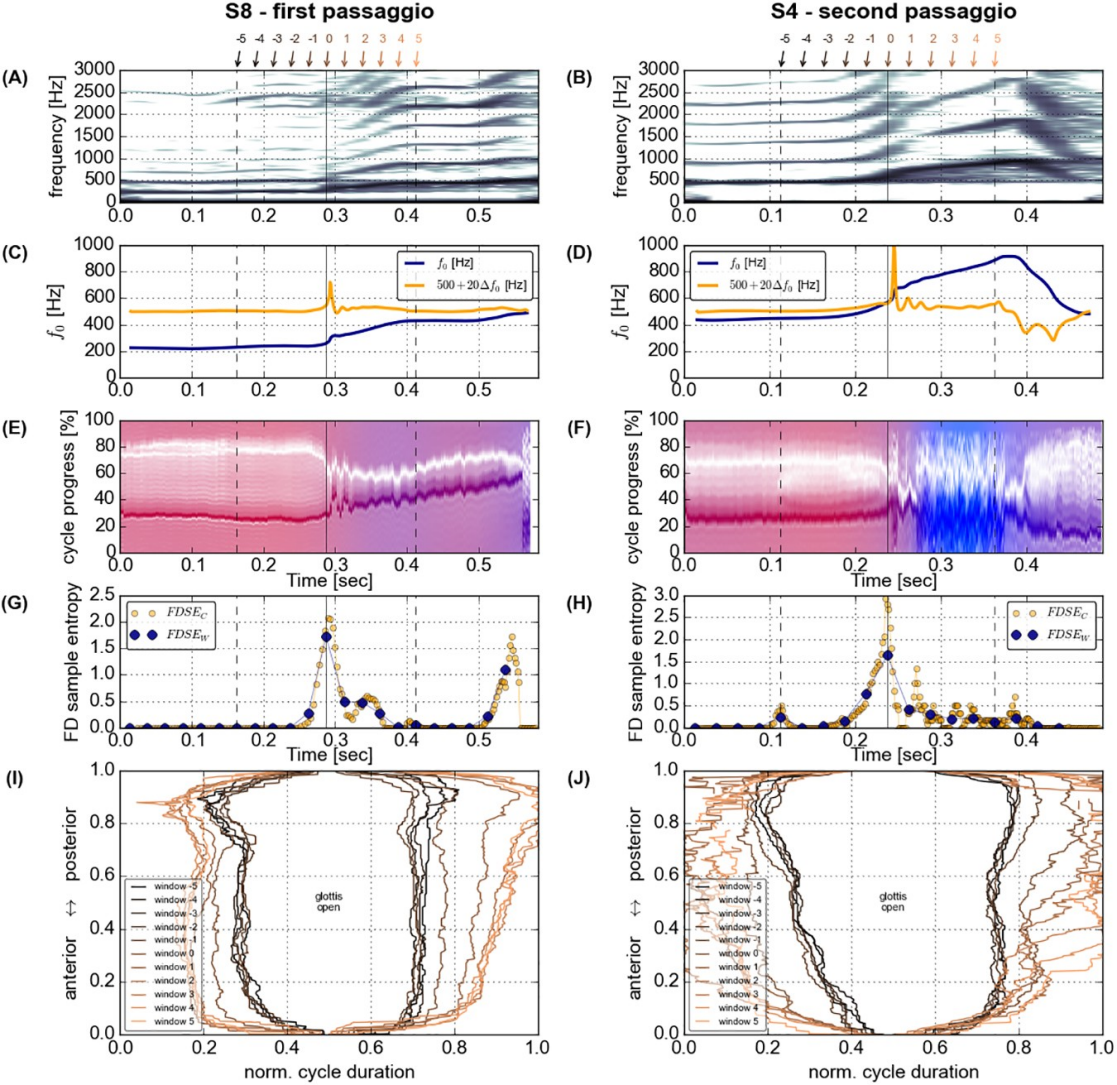
Acoustic spectrogram (window length 1024 frames, 65 dB dynamic range, A and B), time-varying fundamental frequency (*ƒ*_o_ and its first derivative *Δƒ*_o_, C and D), dEGG Wavegram (E and F), cycle based (c) and windows based (w) Fourier Descriptors Sample Entropy (FDSE) (G and H), and summary of glottal opening and closure patterns (I and J) for participants S8 (first *passaggio*) and S4 (second *passaggio*).

Surprisingly, no common laryngeal behavior could be found for the participants’ transitions through either the first or the second *passaggio*. When assessing the dEGG wavegrams and the vocal fold vibration patterns through analysis windows -5 to 5 (S1–S10 Figs), four main strategies emerged:

1. smooth transitions from the lower to the upper pitch, typically coinciding with a **decrease** of the relative duration of vocal fold closure, most prominently seen in participant S4, first *passaggio* (see Fig 1, left panels);
2. smooth transitions with an **increase** of vocal fold contact and closure duration, most prominently seen in participant S1, second *passaggio* (see Fig 1, right panels);
3. abrupt transitions from the lower to the upper pitch coinciding with an abrupt reduction of relative vocal fold closure and contact, most prominently seen in participant S8, first *passaggio* (see Fig 2, left panels); and
4. intermediate episodes of loss of vocal fold contact, most prominently seen in participant S4, second *passaggio* (see Fig 2, right panels)

Summary graphs of all phonations of all participants are included as supplementary material S1–S10 Figs. In these summary graphs, a spectrogram of the acoustic signal (window length 1024 frames, 65 dB dynamic range), the time-varying *ƒ*_o_ and its rate of change, a dEGG Wavegram [62] (see supplementary material S2 Text), both the FDSE_c_ and the FDSE_w_, and a summary of glottal opening and closing patterns are shown for each phonation. Initial data assessment suggests inhomogeneous task execution by the participants. Pitch glides produced with continuous development of laryngeal dynamics (see Fig 1 for two stereotypic examples) were contrasted by pitch glides produced with abrupt changes in laryngeal oscillation patterns and instabilities of the fundamental frequency (see Fig 2) in both the *passaggi*.

**Fig 3:**
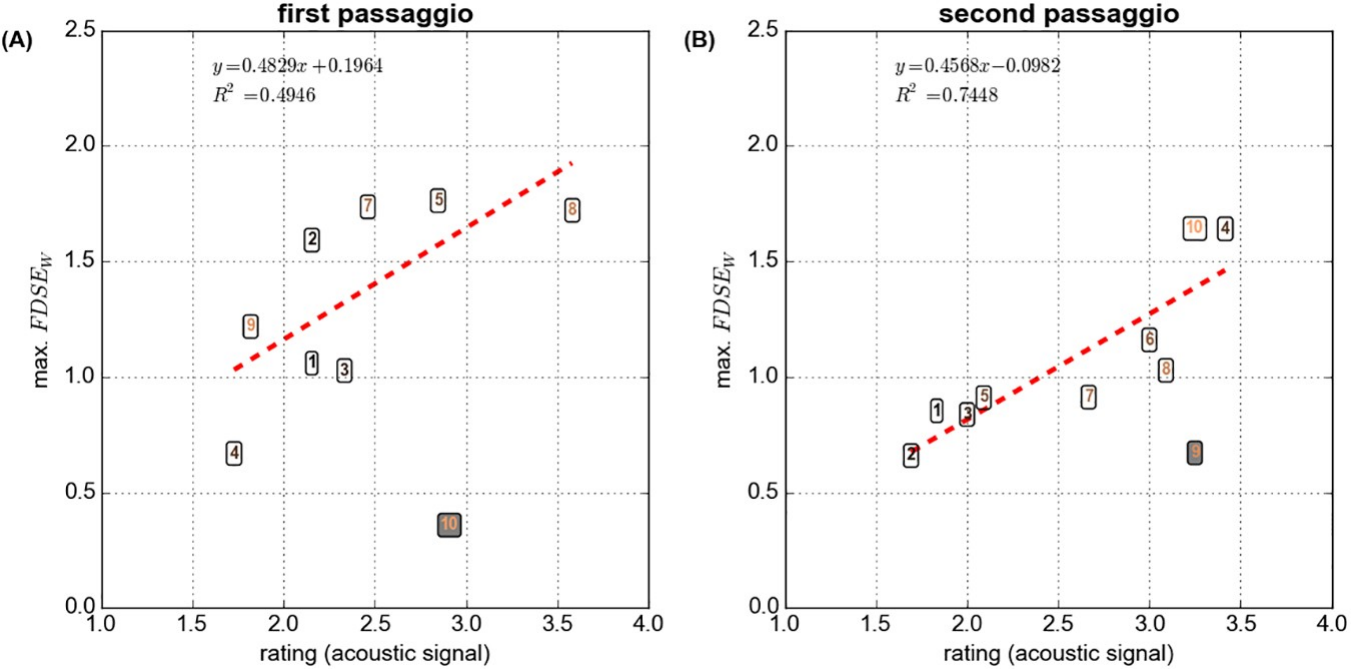
Perceptual rating for the acoustic signal versus the windows based Fourier Descriptors Sample Entropy (FDSE_w_) for the first and second *passaggio*, respectively (A and B). The participant IDs are indicated within the data points. The lines and equations refer to the first order polynomial regression fits.

There was a good agreement between the perceptual rating and the maximum FDSE_w_ (Fig 3). In both the lower (r^2^ = 0.49) and upper pitch glide (r^2^ = 0.74), a higher perceptual rating (indicating a perceptually more prominent registration event) had a tendency to coincide with greater maximum FDSE_w_ (indicating greater alterations of the EGG waveform within an analysis window), and vice-versa.

**Fig 4:**
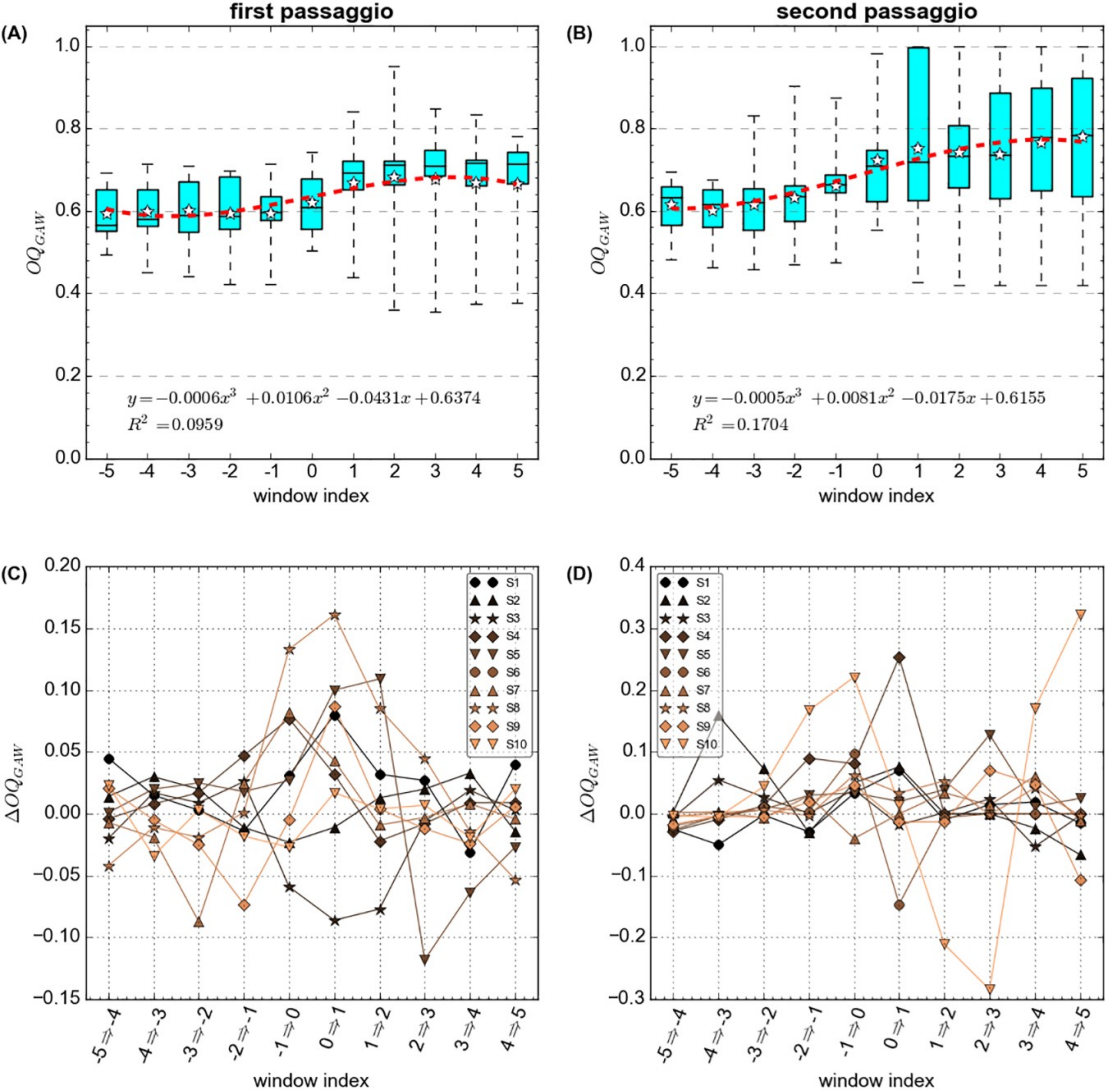
Boxplots for the glottal area waveform (GAW) based open quotient (OQ) in relation to the 25ms windows for the first (A) and second *passaggio* (B). The window 0 refers to the window where the maximum windows based Fourier Descriptors Sample Entropy (FDSE_w_) occurred. Figs C and D show the “between-window” OQ_GAW_ changes for all participants’ first (C) and second *passaggio* (D), respectively.

**Fig 5:**
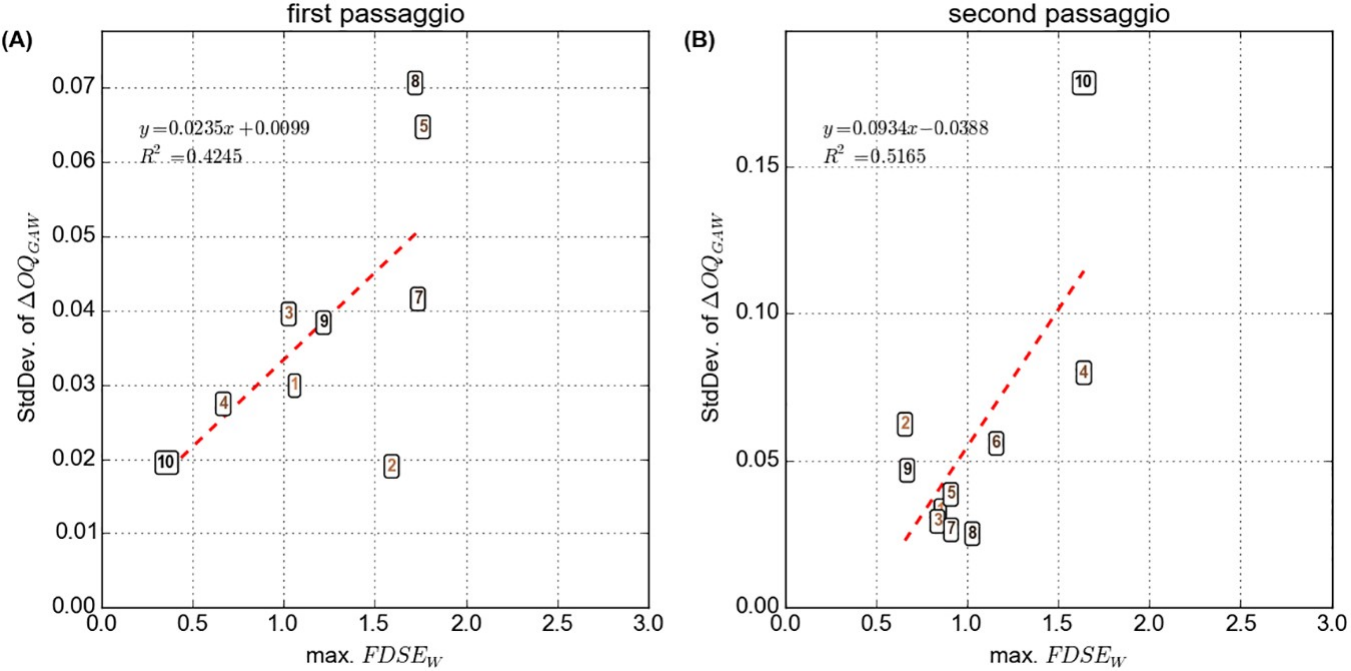
Windows based Fourier Descriptors Sample Entropy (FDSE_w_) versus the standard deviation of the delta open quotient calculated from the glottal area wave form (ΔOQ_GAW_) for the first and second *passaggio*, respectively (A and B). The participant IDs are indicated within the data points. The lines and equations refer to the first order polynomial regression fits.

The mean GAW open quotient (OQ_GAW_), averaged over all participants, increased in both phonation tasks during the course of the pitch glides, by about 10% (lower pitch glide) and 18% (upper pitch glide), respectively (Fig 4A and 4B). In other words, most participants had the tendency to phonate with a longer relative duration of glottal closure per glottal cycle at the beginning of the tasks at lower *ƒ*_o_ as compared to the end of the tasks at higher *ƒ*_o_. In four out of the 20 analyzed phonations, however, the relative duration of vocal fold closure and contact was higher at the end of the respective phonation, as compared to the beginning: S1 (second *passaggio*), S3 (both first and second *passaggio*), S9 (first *passaggio*–see also dEGG wavegrams and vocal fold vibration patterns in the supplementary figures S1 Fig, S3 Fig and S9 Fig). This would explain why a greater variation of OQ_GAW_ values emerged towards end of the tasks within analysis windows 0 to 5, suggesting that the participants utilized different laryngeal strategies for mastering the transitions through their *passaggio* regions, an impression that is also corroborated by inspection of the vibratory patterns–see Discussion. This is also supported by assessment of the OQ_GAW_ differences between consecutive analysis windows in each phonation of the individual participants (ΔOQ_GAW_, Fig 4C and 4D), showing a non-uniform development over the individual analysis windows. Phonations with great ΔOQ_GAW_ “between-window” analysis (e.g., S5 and S8 at the lower pitch glide, or S4 and S10 at the upper pitch glide) had a tendency to coincide with a greater maximum FDSE_w_. This is further illustrated in Fig 5, where the maximum FDSE_w_ is plotted against the standard deviations (across all analysis windows) of the ΔOQ_GAW_ parameter for all participants, showing good correlations between these two parameters in and both phonation tasks and resulting in r^2^ = 0.45 and r^2^ = 0.52, respectively.

## Discussion

In this study transitions through the first and second *passaggi* in a larger number of female singers were analyzed. Despite the fact that all 10 participants were trained classical singers, the results of the perceptual rating suggest that not all phonations were executed without perceptual register transitions, in partial violation of the aesthetical requirement of western classical singing to inaudibly “blend the registers” and to avoid abrupt changes of voice timbre throughout the singing tessitura (i.e., the pitch range used on stage).

The strong correlations between the perceptual rating data, the maximum FDSE_w_, and the “between-window” rate of change of GAW open quotients (recall Figs 3 and 5) suggest that the severity of the perceptual register transition correlated with a vocal fold oscillatory effect: the more audible a register transition, the greater the variations of vocal fold vibration when singing through the *passaggio* region.

Analysis of the dEGG wavegrams and the vocal fold vibration patterns revealed four strategies for navigating the *passaggi* (see Results). Previous research [6,19,47] would support Strategy I, where the relative duration of vocal fold contact and glottal closure would diminish (increasing OQ_GAW_) when increasing *ƒ*_o_, for the first *passaggio*. Thus, the appearance of strategy II in some of the phonations was unexpected. It could be speculated that the increase of relative vocal fold closure duration was induced by an increase of posterior glottal adduction, which was suggested by visual inspection of the respective HSV footage. Such an increased adduction might facilitate the entrainment [63,64] of the two vocal folds in the potentially unstable *passaggio* region, thus helping to stabilize vocal fold vibration.

Phonations utilizing strategy I or II were produced with gradual changes of vocal fold oscillations as *ƒ*_o_ increased. In the first *passaggio*, such gradual changes have already been described using electroglottography in both male [32] and female [62] voices, when performing a register shift from modal to falsetto (for male) or middle (for female) register. The appearance of such gradual changes contradicts the hypothesis [65] that a mixture or blending of the modal and falsetto/middle registers (“voix mixte”) would not be possible when traversing register boundaries in the *passaggio*, i.e., that the voice would be either in modal register (also termed laryngeal mechanism 1 or M1 [65]) or in falsetto/middle register (M2), and that a register transition would always be a distinct and binary event. Yet, our data supports van den Berg’s idea of a “mixture” of modal and falsetto/middle register [13]. The absence of a clear register boundary, as found in our data, calls the definition of registers based on distinct laryngeal mechanisms [66] into question, at least for the professional singers analyzed in this study. Rather, the possibility of gradual adjustments of laryngeal mechanisms might be considered.

Strategy III, resulting in audible registration events and abrupt changes of vocal fold oscillatory patterns, is expected to occur in less proficient singers (see eg. [62], Fig 8). It is therefore not surprising that the clearest emergence of strategy III was found in participant S8, having a rather short period of training and one of the lowest ratings in the Bunch & Chapman taxonomy (see Table 1). Strategy IV involved intermediate loss of vocal fold contact and glottal closure. This could be associated with a slight and sudden abductory gesture of the arytenoids. Given the hypothesis that strategies I and II with a smooth transition could be both associated with gradual abduction or adduction, the contact losses of Strategy IV could reveal a deficit in such coordination. On the other hand, due to limitations in data storage space of the HSV camera, all phonatory tasks in this study had to be performed within one second, with the major *ƒ*_o_ increase typically occurring within intervals of 50 ms to 250 ms. Typically, singers have more time for coordinating their *passaggio* for their performance on stage. Theoretically, the episodes of contact loss seen in strategy IV could therefore also be artifacts introduced by the data acquisition protocol.

The changes in oscillation patterns found in our data are to be expected for the lower pitch glides through the first *passaggio*, i.e. the register transition from modal register to middle register [48] (sometimes also termed M1 and M2, respectively [6,47]). Despite some cases of disagreement concerning changes of vocal fold closure (see above), for this transition, our data corroborates the general previously reported finding of laryngeal adjustments [6,12,47,48]. More surprising, however, is the finding that the transitions through the second *passaggio*, which occurred during the upper pitch glides, also caused considerable variations of vocal fold oscillation patterns. Preliminary evidence for this phenomenon, albeit with limited video frame duration/spatial resolution, has been brought forward in two previous single-subject studies involving two untrained female singers [19,39]. Here we provide the first conclusive confirmation for such vocal fold vibration pattern adjustments through the second *passaggio* in the female voice, utilizing HSV recordings with sufficient temporal and spatial resolution. Our data clearly demonstrates that the female second *passaggio* is not affected by vocal tract resonances alone, that is, without changes of vocal fold oscillations patterns.

The reason for these laryngeal oscillatory changes in the second *passaggio* is, however, unclear. One possibility is that the laryngeal oscillation patterns were caused by changes of laryngeal muscle activity. A second hypothesis, mainly based on theoretical modeling, suggests that the supraglottal vocal tract can interact with vocal fold oscillation patterns (level 2 interactions according to Titze [67]) and that voice instabilities could be expected when *ƒ*_o_ or an integer multiple of it (i.e, a harmonic), is at or above the first vocal tract resonance (*ƒ*_R1_) [67,68]. In the vowel [iː], used for the phonations analyzed in this study, *ƒ*_R1_ is typically found around 350Hz [8,69,70], at least in speech. In trained classical singing, *ƒ*_R1_ is customarily raised together with *ƒ*_o_ when *ƒ*_o_ is close to *ƒ*_R1_ [20,51,52], presumably in order to avoid a crossing of *ƒ*_o_ and *ƒ*_R1_ and the expected voice instabilities associated with this situation. Theory predicts that whenever a resonance is close to a harmonic, non-linear interactions between the inert vocal tract and the voice source might occur, either desirable (when the respective harmonic is just below the resonance), or undesirable (when the harmonic is at or slightly above the resonance) [67]. In this light, the changes of vocal fold vibration seen in the second *passaggio* could very well be induced by non-linear interactions between the vocal tract and the sound source. However, as our experimental setup did not allow for measurement of the supraglottal vocal tract resonances, this hypothesis could neither be confirmed nor ruled out.

There are some important limitations of this study. The participant pool was limited to 10 professionally trained sopranos. It could thus not be ruled out that other singers would use different strategies for navigating through the *passaggi*. Also, the number of subjects might have been too small to verify if, and to what extent, the described four laryngeal strategies for traversing the *passagio* zones are relevant. On the other hand, it is hardly feasible to include a greater number of professional singers for such an invasive experimental study. Secondly, the experiment only considers ascending glides. In experiments on male voices it has been shown that an ascending glide across the *passaggio* showed greater irregularities of vocal fold oscillations than descendent glides and that the *ƒ*_o_ of the *passaggio* was found lower for the descendent glide [71], resulting in a hysteresis. As a consequence, it might be possible that register transitions on descending glides would reveal different strategies. Thirdly, it cannot be excluded that some of the perceptually noticeable register transitions were also influenced by the artificial recording situation (endoscope in the nose). However, the flexible endoscope approach was preferred over a rigid endoscope since it allows for more natural phonation. Though some subjects might not have executed the tasks to the best of their ability (as they would on stage), the recorded phonations are not artifacts. Rather, they constitute valuable data in the sense that they are examples of how the larynx can behave during transitions through either *passaggio*. Lastly, the study is only concerned with western classical singers. A wide variety of other singing styles exists, in which different strategies for navigating the *passaggi* exist, such as musical theater singing, pop/rock singing or yodeling. Analysis of such important groups of singers is left to future investigations.

## Conclusions

This study provides evidence of vocal fold oscillatory changes during the first and second *passaggi* in a larger number of female singers. It is the first of its kind to utilize laryngeal imaging with sufficient temporal and spatial resolution. The findings suggest that noteworthy vocal fold oscillatory registration events occur even in professional (trained) singers. Four different laryngeal strategies were found for navigating the *passaggio* regions: smooth transitions with increasing or decreasing durations of glottal closure, abrupt register transitions, and intermediate loss of vocal fold contact, possibly accompanied by abductory gestures in the larynx. Audible register transitions (in both the first and second *passaggio*) were accompanied by noteworthy changes of vocal fold vibration patterns. This would suggest that either (a) the respective transitions were caused by the sound source through changing laryngeal biomechanical properties induced by intrinsic laryngeal muscles, or that (b) occurring vocal tract resonance effects had a strong influence on the sound source as described by Titze’s level 2 interactions [67]. Further research is necessary to identify which of these two hypotheses is applicable.

## Supporting information

S1 Text Sample Entropy Calculation. (DOCX)

S2 Text Wavegram generation. (DOCX)

S1 Fig Vibration Synopsis for subject S1. Acoustic spectrogram (window length 1024 frames, 65 dB dynamic range, A and B), time-varying fundamental frequency (*ƒ*_o_, C and D), dEGG Wavegram (E and F), cycle based (c) and windows based (w) Fourier Descriptors Sample Entropy (FDSE) (G and H), and summary of glottal opening and closure patterns (I and J) for subject 1.
(TIF)

S2 Fig Vibration Synopsis for subject S2. Acoustic spectrogram (window length 1024 frames, 65 dB dynamic range, A and B), time-varying fundamental frequency (*ƒ*_o_, C and D), dEGG Wavegram (E and F), cycle based (c) and windows based (w) Fourier Descriptors Sample Entropy (FDSE) (G and H), and summary of glottal opening and closure patterns (I and J) for subject 2.
(TIF)

S3 Fig Vibration Synopsis for subject S3. Acoustic spectrogram (window length 1024 frames, 65 dB dynamic range, A and B), time-varying fundamental frequency (*ƒ*_o_, C and D), dEGG Wavegram (E and F), cycle based (c) and windows based (w) Fourier Descriptors Sample Entropy (FDSE) (G and H), and summary of glottal opening and closure patterns (I and J) for subject 3.
(TIF)

S4 Fig Vibration Synopsis for subject S4. Acoustic spectrogram (window length 1024 frames, 65 dB dynamic range, A and B), time-varying fundamental frequency (*ƒ*_o_, C and D), dEGG Wavegram (E and F), cycle based (c) and windows based (w) Fourier Descriptors Sample Entropy (FDSE) (G and H), and summary of glottal opening and closure patterns (I and J) for subject 4.
(TIF)

S5 Fig Vibration Synopsis for subject S5. Acoustic spectrogram (window length 1024 frames, 65 dB dynamic range, A and B), time-varying fundamental frequency (*ƒ*_o_, C and D), dEGG Wavegram (E and F), cycle based (c) and windows based (w) Fourier Descriptors Sample Entropy (FDSE) (G and H), and summary of glottal opening and closure patterns (I and J) for subject 5.
(TIF)

S6 Fig Vibration Synopsis for subject S6. Acoustic spectrogram (window length 1024 frames, 65 dB dynamic range, A and B), time-varying fundamental frequency (*ƒ*_o_, C and D), dEGG Wavegram (E and F), cycle based (c) and windows based (w) Fourier Descriptors Sample Entropy (FDSE) (G and H), and summary of glottal opening and closure patterns (I and J) for subject 6.
(TIF)

S7 Fig Vibration Synopsis for subject S7. Acoustic spectrogram (window length 1024 frames, 65 dB dynamic range, A and B), time-varying fundamental frequency (*ƒ*_o_, C and D), dEGG Wavegram (E and F), cycle based (c) and windows based (w) Fourier Descriptors Sample Entropy (FDSE) (G and H), and summary of glottal opening and closure patterns (I and J) for subject 7.
(TIF)

S8 Fig Vibration Synopsis for subject S8. Acoustic spectrogram (window length 1024 frames, 65 dB dynamic range, A and B), time-varying fundamental frequency (*ƒ*_o_, C and D), dEGG Wavegram (E and F), cycle based (c) and windows based (w) Fourier Descriptors Sample Entropy (FDSE) (G and H), and summary of glottal opening and closure patterns (I and J) for subject 8.
(TIF)

S9 Fig Vibration Synopsis for subject S9. Acoustic spectrogram (window length 1024 frames, 65 dB dynamic range, A and B), time-varying fundamental frequency (*ƒ*_o_, C and D), dEGG Wavegram (E and F), cycle based (c) and windows based (w) Fourier Descriptors Sample Entropy (FDSE) (G and H), and summary of glottal opening and closure patterns (I and J) for subject 9.
(TIF)

S10 Fig Vibration Synopsis for subject S10. Acoustic spectrogram (window length 1024 frames, 65 dB dynamic range, A and B), time-varying fundamental frequency (*ƒ*_o_, C and D), dEGG Wavegram (E and F), cycle based (c) and windows based (w) Fourier Descriptors Sample Entropy (FDSE) (G and H), and summary of glottal opening and closure patterns (I and J) for subject 10.
(TIF)

S11 Fig Basic processing steps of wavegram creation (see S2 Text). The two halves of the image illustrate the creation of wavegrams based on the EGG (left) and the dEGG signal (right), respectively.
(TIF)

S1 Excel Raw data for Fig 3. (CSV)

S2 Excel Raw data for Fig 4. (CSV)

S3 Excel Raw data for Fig 5. (CSV)

## References

[1] Titze IR (1994) Principles of voice production Prentice Hall, NJ.

[2] Lehfeldt C (1835) Nonulla de vocis formatione Berlin, cited by Müller J: Handbuch der Physiologie des Menschen für Vorlesungen.Coblenz,Verlag von J.Hölscher,1840: Inauguraldissertation.

[3] Müller J (1840) Handbuch der Physiologie des Menschen für Vorlesungen. Coblenz: Verlag von J. Hölscher.

[4] Merkel CL (1863) Anatomie und Physiologie des menschlichen Stimm- und Sprachorgans (Anthropophonik). Leipzig: Ambrosius Abel Verlag.

[5] Nadoleczny M (1923) Untersuchungen über den Kunstgesang. Berlin: Springer.

[6] Henrich N (2006) Mirroring the voice from Garcia to the present day: some insights into singing voice registers. Logoped Phoniatr Vocol 31: 3–14. doi: 10.1080/14015430500344844 16531287 10.1080/14015430500344844

[7] Hollien H (1983) Report on vocal registers In: A A, F S, J E, S J, editors. Stockholm Musical Acoustic Conference (SMAC). 46 ed. Stockholm: Royal Swedish Academy of Music pp. 27–35.

[8] Sundberg J (1987) The Science of the Singing Voice: Northern Illinois University Press, Dekalb, IL, USA.

[9] Large J (1972) Towards an Integrated Physiologic-Acoustic Theory of Vocal Registers. The NATS Bulletin 28: 18–36.

[10] Kochis-Jennings KA, Finnegan EM, Hoffman HT, Jaiswal S (2012) Laryngeal muscle activity and vocal fold adduction during chest, chestmix, headmix, and head registers in females. J Voice 26: 182–193. doi: 10.1016/j.jvoice.2010.11.002 21596521 10.1016/j.jvoice.2010.11.002

[11] Echternach M (2010) Untersuchungen zu Registerübergängen bei männlichen Stimmen Bochum: Projektverlag.

[12] Miller DG (2000) Registers in singing: empirical and systematic studies in the theory of the singing voice Groningen: University of Groningen.

[13] Van den Berg JW (1963) Vocal ligaments versus registers. NATS Bulletin 20: 16–21.

[14] Miller R (1986) The Structure of Singing. New York: Schirmer Books.

[15] Vennard WL (1967) Singing: the mechanism and the technic New York: Carl Fischer.

[16] Hirano M, Vennard W, Ohala J (1970) Regulation of register, pitch and intensity of voice. An electromyographic investigation of intrinsic laryngeal muscles. Folia Phoniatr (Basel) 22: 1–20. 5430060 10.1159/000263363

[17] Neumann K, Schunda P, Hoth S, Euler HA (2005) The interplay between glottis and vocal tract during the male passaggio. Folia Phoniatr Logop 57: 308–327. doi: 10.1159/000087084 16280632 10.1159/000087084

[18] Echternach M, Sundberg J, Baumann T, Markl M, Richter B (2011) Vocal tract area functions and formant frequencies in opera tenors' modal and falsetto registers. J Acoust Soc Am 129: 3955–3963. doi: 10.1121/1.3589249 21682417 10.1121/1.3589249

[19] Garnier M, Henrich N, Crevier-Buchman L, Vincent C, Smith J, et al (2012) Glottal behavior in the high soprano range and the transition to the whistle register. J Acoust Soc Am 131: 951–962. doi: 10.1121/1.3664008 22280718 10.1121/1.3664008

[20] Henrich N, Smith J, Wolfe J (2011) Vocal tract resonances in singing: Strategies used by sopranos, altos, tenors, and baritones. J Acoust Soc Am 129: 1024–1035. doi: 10.1121/1.3518766 21361458 10.1121/1.3518766

[21] Titze IR (1988) A framework for the study of vocal registers. J Voice 2: 183–194.

[22] Titze IR (2014) Bi-stable vocal fold adduction: a mechanism of modal-falsetto register shifts and mixed registration. J Acoust Soc Am 135: 2091–2101. doi: 10.1121/1.4868355 25235006 10.1121/1.4868355 PMC4167751

[23] Zanartu M, Mehta DD, Ho JC, Wodicka GR, Hillman RE (2011) Observation and analysis of in vivo vocal fold tissue instabilities produced by nonlinear source-filter coupling: a case study. J Acoust Soc Am 129: 326–339. doi: 10.1121/1.3514536 21303014 10.1121/1.3514536 PMC3055289

[24] Tokuda IT, Zemke M, Kob M, Herzel H (2010) Biomechanical modeling of register transitions and the role of vocal tract resonators. J Acoust Soc Am 127: 1528–1536. doi: 10.1121/1.3299201 20329853 10.1121/1.3299201

[25] Titze IR, Worley AS (2009) Modeling source-filter interaction in belting and high-pitched operatic male singing. J Acoust Soc Am 126: 1530 doi: 10.1121/1.3160296 19739766 10.1121/1.3160296 PMC2757425

[26] Colton RH (1972) Spectral characteristics of the modal and falsetto registers. Folia Phoniatr (Basel) 24: 337–344. 4670039 10.1159/000263588

[27] Colton RH (1973) Vocal intensity in the modal and falsetto registers. Folia Phoniatr (Basel) 25: 62–70. 4700764 10.1159/000263671

[28] Sundberg J, Högset C (2001) Voice source differences between falsetto and modal registers in counter tenors tenors and baritons. Logoped Phoniatr Vocol 26: 26–36. 11432412

[29] Roubeau B, Henrich N, Castellengo M (2009) Laryngeal Vibratory Mechanisms: The Notion of Vocal Register Revisited. J Voice 23: 425–438. doi: 10.1016/j.jvoice.2007.10.014 18538982 10.1016/j.jvoice.2007.10.014

[30] Echternach M, Sundberg J, Zander MF, Richter B (2011) Perturbation measurements in untrained male voices' transitions from modal to falsetto register. J Voice 25: 663–669. doi: 10.1016/j.jvoice.2010.01.013 20488660 10.1016/j.jvoice.2010.01.013

[31] Svec JG, Schutte HK, Miller DG (1999) On pitch jumps between chest and falsetto registers in voice: data from living and excised human larynges. J Acoust Soc Am 106: 1523–1531. 10489708 10.1121/1.427149

[32] Echternach M, Traser L, Richter B (2012) Perturbation of Voice Signals in Register Transitions on Sustained Frequency in Professional Tenors. J Voice 26: 674.e615. 10.1016/j.jvoice.2012.02.003 22633333

[33] Echternach M, Dippold S, Richter B (2016) High-speed imaging using rigid laryngoscopy for the analysis of register transitions in professional operatic tenors. Logoped Phoniatr Vocol 41: 1–8. doi: 10.3109/14015439.2014.936499 25017997 10.3109/14015439.2014.936499

[34] Echternach M, Traser L, Markl M, Richter B (2011) Vocal tract configurations in male alto register functions. J Voice 25: 670–677. doi: 10.1016/j.jvoice.2010.09.008 21402469 10.1016/j.jvoice.2010.09.008

[35] Echternach M, J. S, Arndt S, Breyer T, M. M, et al (2008) Vocal Tract and Register Changes Analysed by Real Time MRI in Male Professional Singers–a Pilot Study. Logoped Phoniatr Vocol 33: 67–73. doi: 10.1080/14015430701875653 18569645 10.1080/14015430701875653

[36] Echternach M (2010) Untersuchung zur Analyse der Stimmlippenschwingungen bei professionellen Tenören mittels Hochgeschwindigkeitsglottographie Untersuchungen zu Registerübergängen bei männlichen Stimmen. Bochum: Projektverlag pp. 97–104.

[37] Echternach M, Traser L, Richter B (2014) Vocal Tract Configurations in Tenors' Passaggio in Different Vowel Conditions-A Real-Time Magnetic Resonance Imaging Study. J Voice 28:262e1–e8. 10.1016/j.jvoice.2013.10.009 24412038

[38] Miller DG, Schutte HK (1993) Physical definition of the "flageolet register". J Voice 7: 206–212. 8353637 10.1016/s0892-1997(05)80328-5

[39] Svec JG, Sundberg J, Hertegard S (2008) Three registers in an untrained female singer analyzed by videokymography, strobolaryngoscopy and sound spectrography. J Acoust Soc Am 123: 347–353. doi: 10.1121/1.2804939 18177164 10.1121/1.2804939

[40] Echternach M, Sundberg J, Arndt S, Markl M, Schumacher M, et al (2010) Vocal tract in female registers–a dynamic real-time MRI study. J Voice 24: 133–139. doi: 10.1016/j.jvoice.2008.06.004 19185452 10.1016/j.jvoice.2008.06.004

[41] Sonninen A, Hurme P, Laukkanen AM (1999) The external frame function in the control of pitch, register, and singing mode: radiographic observations of a female singer. J Voice 13: 319–340. 10498050 10.1016/s0892-1997(99)80039-3

[42] Herbst CT, Qiu Q, Schutte HK, Svec JG (2011) Membranous and cartilaginous vocal fold adduction in singing. J Acoust Soc Am 129: 2253–2262. doi: 10.1121/1.3552874 21476680 10.1121/1.3552874

[43] Garnier M, Henrich N, Smith J, Wolfe J (2010) Vocal tract adjustments in the high soprano range. J Acoust Soc Am 127: 3771–3780. doi: 10.1121/1.3419907 20550275 10.1121/1.3419907

[44] Echternach M, Birkholz P, Traser L, Fluegge TV, Kamberger R, et al (2015) Articulation and vocal tract acoustics at soprano subject's high fundamental frequencies. Journal of the Acoustical Society of America 137: 2586–2595. doi: 10.1121/1.4919356 25994691 10.1121/1.4919356

[45] Sundberg J, Kullberg A (1999) Voice source studies of register differences in untrained female singing. Logop Phon Vocol 24: 76–83.

[46] Large J (1968) An acoustical study of isoparametric tones in the chest and middle registers in inging. NATS Bulletin 24: 12–15.

[47] Roubeau B, Henrich N, Castellengo M (2009) Laryngeal vibratory mechanisms: the notion of vocal register revisited. J Voice 23: 425–438. doi: 10.1016/j.jvoice.2007.10.014 18538982 10.1016/j.jvoice.2007.10.014

[48] Rubin HJ, Hirt CC (1960) The falsetto. A high speed cinematographic study. Laryngoscope 70: 1305–1324. doi: 10.1288/00005537-196009000-00008 13744357 10.1288/00005537-196009000-00008

[49] Sundberg J (2009) Articulatory configuration and pitch in a classically trained soprano singer. J Voice 23: 546–551. doi: 10.1016/j.jvoice.2008.02.003 18504111 10.1016/j.jvoice.2008.02.003

[50] Bresch E, Narayanan S (2010) Real-time magnetic resonance imaging investigation of resonance tuning in soprano singing. J Acoust Soc Am 128: EL335–EL341. doi: 10.1121/1.3499700 21110548 10.1121/1.3499700 PMC2997814

[51] Sundberg J (1975) Formant technique in a professional female singer. Acustica 32: 89–96.

[52] Joliveau E, Smith J, Wolfe J (2004) Acoustics: tuning of vocal tract resonance by sopranos. Nature 427: 116 doi: 10.1038/427116a 14712266 10.1038/427116a

[53] Svec JG, Schutte HK (1996) Videokymography: high-speed line scanning of vocal fold vibration. J Voice 10: 201–205. 8734395 10.1016/s0892-1997(96)80047-6

[54] Bunch M, Chapman J (2000) Taxonomy of singers used as subjects in scientific research. J voice 14: 363–369. 11021503 10.1016/s0892-1997(00)80081-8

[55] Seidner W, Wendler J (2004) Die Sängerstimme. Berlin: Henschel Verlag.

[56] Echternach M, Burk F, Köberlein M, Herbst CT, Döllinger M, et al (2016) Oscillatory characteristics of the vocal folds across the tenor passaggio J Voice: Epub ahead of print August 6th 2016. 10.1016/j.jvoice.2016.06.015 27499033

[57] Bruton A, Conway J, Holgate ST (2000) Reliability: What is it and how is it measured? Physiotherapy 86: 94–99.

[58] Inwald EC, Dollinger M, Schuster M, Eysholdt U, Bohr C (2011) Multiparametric analysis of vocal fold vibrations in healthy and disordered voices in high-speed imaging. J Voice 25: 576–590. doi: 10.1016/j.jvoice.2010.04.004 20728308 10.1016/j.jvoice.2010.04.004

[59] Hampala V, Garcia M, Svec JG, Scherer RC, Herbst CT (2016) Relationship Between the Electroglottographic Signal and Vocal Fold Contact Area. J Voice 30:161–171. doi: 10.1016/j.jvoice.2015.03.018 26256493 10.1016/j.jvoice.2015.03.018

[60] Selamtzis A, Ternström S (2014) Analysis of vibratory states in phonation using spectral features of the electroglottographic signal. J Acoust Soc Am 136: 2773–2783. doi: 10.1121/1.4896466 25373977 10.1121/1.4896466

[61] Richman JS, Moorman JR (2000) Physiological time-series analysis using approximate entropy and sample entropy. Am J Physiol Heart Circ Physiol 278: H2039–2049. 10843903 10.1152/ajpheart.2000.278.6.H2039

[62] Herbst CT, Fitch WT, Svec JG (2010) Electroglottographic wavegrams: a technique for visualizing vocal fold dynamics noninvasively. J Acoust Soc Am 128: 3070–3078. doi: 10.1121/1.3493423 21110602 10.1121/1.3493423

[63] Berry D (2001) Mechanism of modal and non-modal phonation. J Phon 29: 431–450.

[64] Berke GS, Gerratt BR (1993) Laryngeal biomechanics: an overview of mucosal wave mechanics. J Voice 7: 123–128. 8353625 10.1016/s0892-1997(05)80341-8

[65] Castellengo M, Chuberre B, Henrich N (2004) Is voix mixte, the vocal technique use to smoothe the transition across the two main laryngeal mechanisms, an independent mechanism? Proceedings of the International Symposium on Musical Acoustics.

[66] Garcia M (1841) Memoire sur la voix humaine. L`Esculape 3: 105.

[67] Titze IR (2008) Nonlinear source-filter coupling in phonation: theory. J Acoust Soc Am 123: 2733–2749. doi: 10.1121/1.2832337 18529191 10.1121/1.2832337 PMC2811547

[68] Titze IR, Baken RJ, Bozeman KW, Granqvist S, Henrich N, et al (2015) Toward a consensus on symbolic notation of harmonics, resonances, and formants in vocalization. J Acoust Soc Am 137: 3005–3007. doi: 10.1121/1.4919349 25994732 10.1121/1.4919349 PMC5392060

[69] Fant G (1960) Acoustic Theory of Speech Production. The Hague: Mouton.

[70] Peterson GE, Barney HL (1952) Control methods used in study of the vowels. J Acoust Soc Am 24: 175–184.

[71] Echternach M, Richter B (2012) Passaggio in the professional tenor voice–evaluation of perturbation measures. J Voice 26: 440–446. doi: 10.1016/j.jvoice.2011.03.004 21550773 10.1016/j.jvoice.2011.03.004

