# Supplementary material for: Laryngeal evidence for the first and second passaggio in professionally trained sopranos
Source: PLoS One. 2017 May 3;12(5):e0175865. doi: 10.1371/journal.pone.0175865 (PMC5414960; doi:10.1371/journal.pone.0175865)
Supplement: S1 Text — (DOCX) [file pone.0175865.s001.docx]

Sample entropy calculation

For marking abrupt transitions in the EGG signal we calculated a Sample Entropy (SE) based marker in a cycle-synchronous fashion, using the sampen algorithm by Richman and Moorman (2000) [1] as provided in the website by these authors (https://www.physionet.org/physiotools/sampen/matlab/1.1-1).

The EGG signal of each analyzed phonation was bandpassed with a 3rd order Butterworth filter (cutoff frequencies 80 Hz and 5000 Hz), using zero-phase filtering, as implemented by MATLAB’s function filtfilt. Since the signal was filtered two times with this implementation, the effective order of the filter became 6.

The EGG signal was separated into cycles, and the amplitudes and phases of the first two harmonics were extracted by computing the DFT of each cycle. These four cycle-synchronous time series (two for the amplitudes and two for the phases) were then used separately as input to the sampen algorithm. Before being used as input, each amplitude time series was normalized to unity, while each phase time series was divided by 2 π times its corresponding number of harmonic (1 for the first, 2 for the second).

The SE was computed as the sum of the “partial” SE values, i.e., one for each of the four time series, as follows. Each partial SE time series had a value for each vibratory cycle, which was computed by calculating the SE for a window of cycles centered at the cycle of interest. Typically used values for the window were 10 cycles with 9 cycles overlap. The epoch length was m=1 for all four time series and the matching tolerance was chosen to be r=0.1 for the magnitudes and r=0.4 for the phases. This resulted in 4 SE time-series. The final SE-based marker was obtained by summing the four partial SE values.

These steps were repeated n times for each EGG signal (where n is the number of EGG glottal cycles in each respective signal), resulting in a series of time-varying SE values for each analyzed EGG signal.
