# Supplementary material for: Laryngeal evidence for the first and second passaggio in professionally trained sopranos
Source: PLoS One. 2017 May 3;12(5):e0175865. doi: 10.1371/journal.pone.0175865 (PMC5414960; doi:10.1371/journal.pone.0175865)
Supplement: S2 Text — (DOCX) [file pone.0175865.s002.docx]

Wavegram generation

dEGG wavegram generation is outlined in [2]. In short, is achieved in three steps: (1) the dEGG signal (i.e., the first mathematical derivative of the EGG signal) is decomposed into individual cycles, each normalized in both duration and amplitude; (2) the amplitude within each cycle is color coded; and (3) the resulting strips of pixels are consecutively displayed from bottom to top along the y-axis, where the position on the x-axis represents the respective time coordinate (ie, overall time is mapped onto the x-axis). This process is illustrated in supplementary Fig S11 below. The wavegram display has been found to be useful in visualizing the change of both vocal register and the degree of vocal fold adduction [2].

References

[1] Richman, Joshua S., and J. Randall Moorman. "Physiological time-series analysis using approximate entropy and sample entropy." American Journal of Physiology-Heart and Circulatory Physiology 278.6 (2000): H2039-H2049.

[2] Herbst CT, Fitch WT, Svec JG (2010) Electroglottographic wavegrams: a technique for visualizing vocal fold dynamics noninvasively. J Acoust Soc Am 128: 3070-3078.

[3] Christian T. Herbst, Markus Hess, Frank Müller, Jan G. Svec, Johan Sundberg (2015). Glottal adduction and subglottal pressure in singing. Journal of Voice, 29 (4), 391-402.
