# Supplementary figures and images for: Laryngeal evidence for the first and second passaggio in professionally trained sopranos
Source: PLoS One. 2017 May 3;12(5):e0175865. doi: 10.1371/journal.pone.0175865 (PMC5414960; doi:10.1371/journal.pone.0175865)

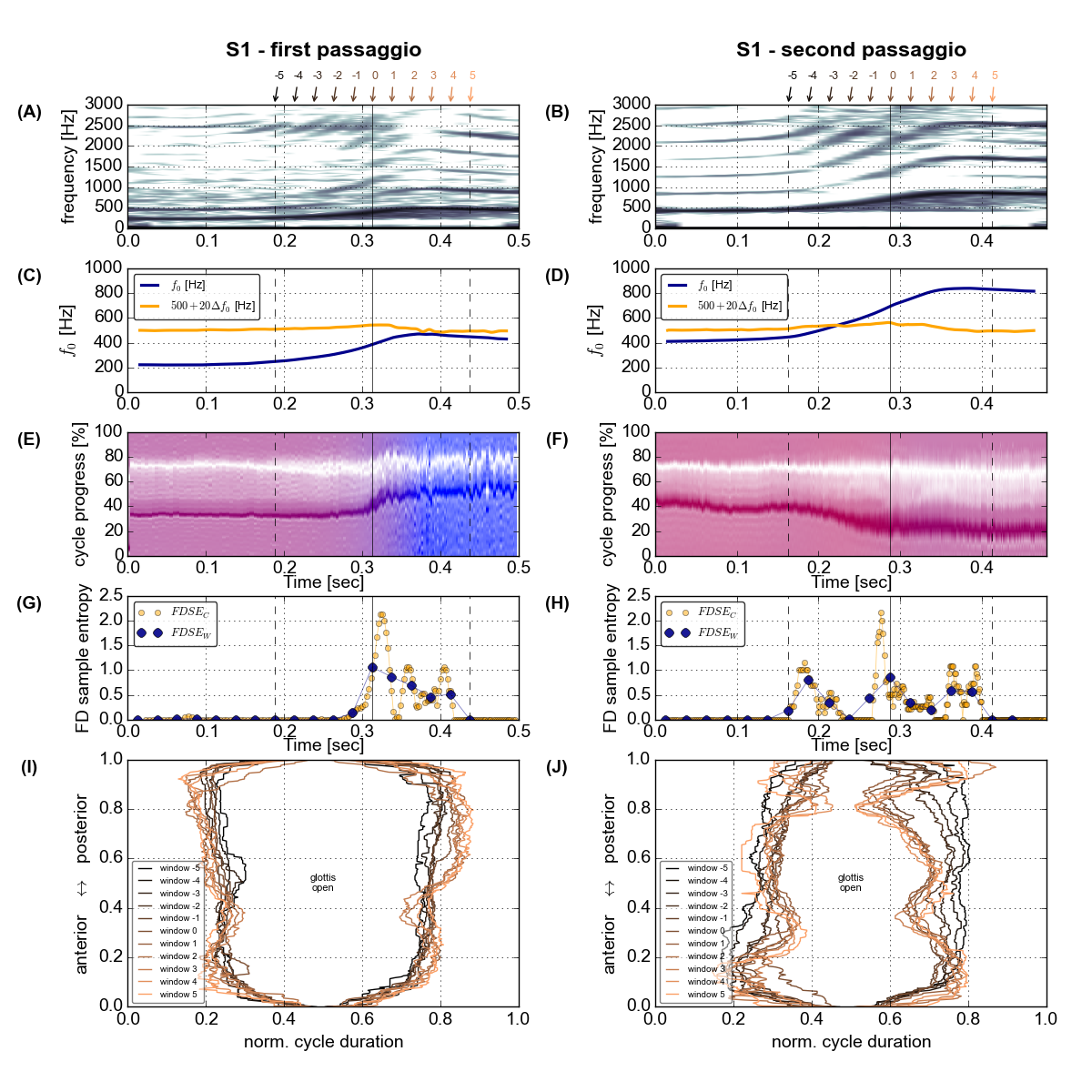

Supplement: S1 Fig — Acoustic spectrogram (window length 1024 frames, 65 dB dynamic range, A and B), time-varying fundamental frequency (ƒo, C and D), dEGG Wavegram (E and F), cycle based (c) and windows based (w) Fourier Descriptors Sample Entropy (FDSE) (G and H), and summary of glottal opening and closure patterns (I and J) for subject 1. (TIF) [file pone.0175865.s003.tif]

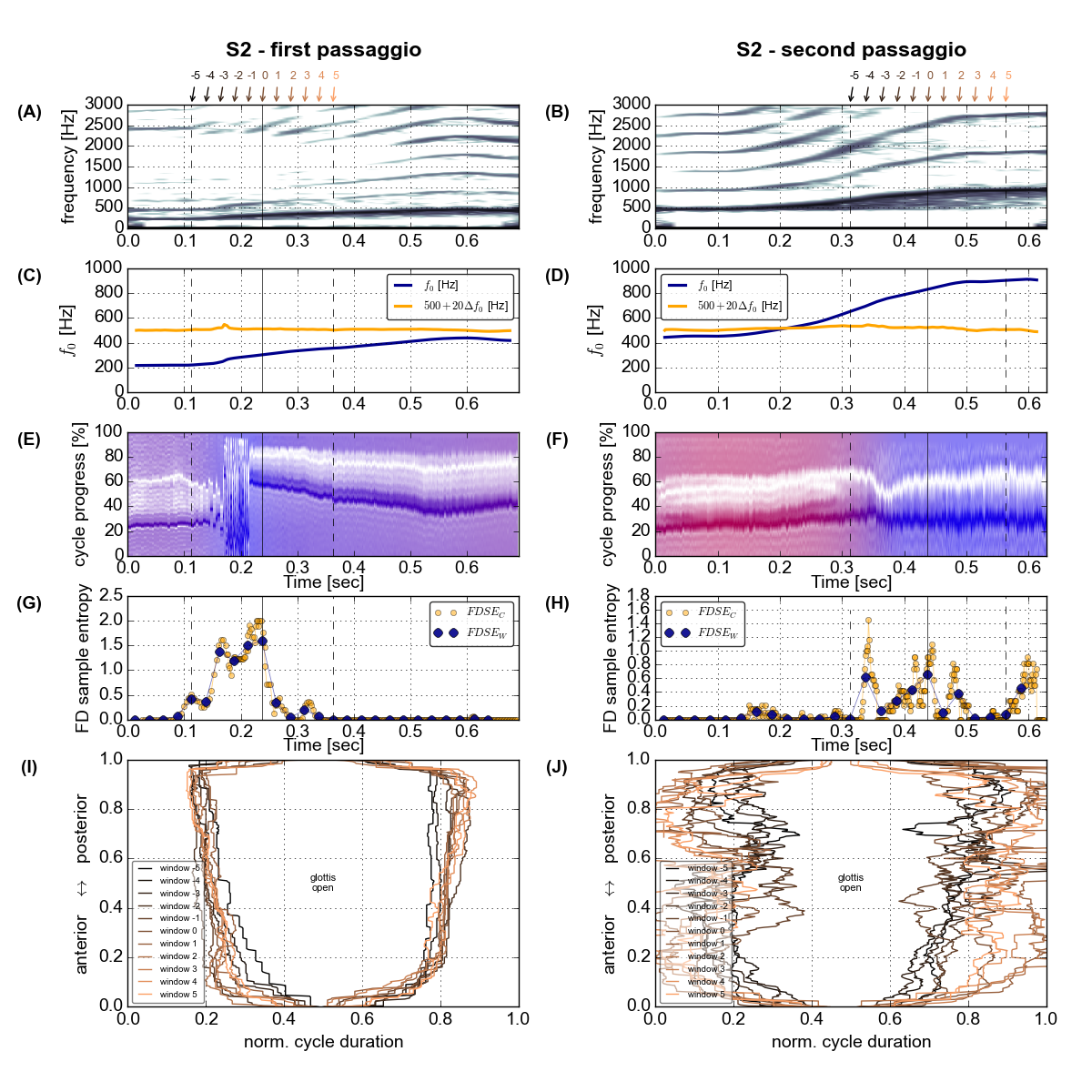

Supplement: S2 Fig — Acoustic spectrogram (window length 1024 frames, 65 dB dynamic range, A and B), time-varying fundamental frequency (ƒo, C and D), dEGG Wavegram (E and F), cycle based (c) and windows based (w) Fourier Descriptors Sample Entropy (FDSE) (G and H), and summary of glottal opening and closure patterns (I and J) for subject 2. (TIF) [file pone.0175865.s004.tif]

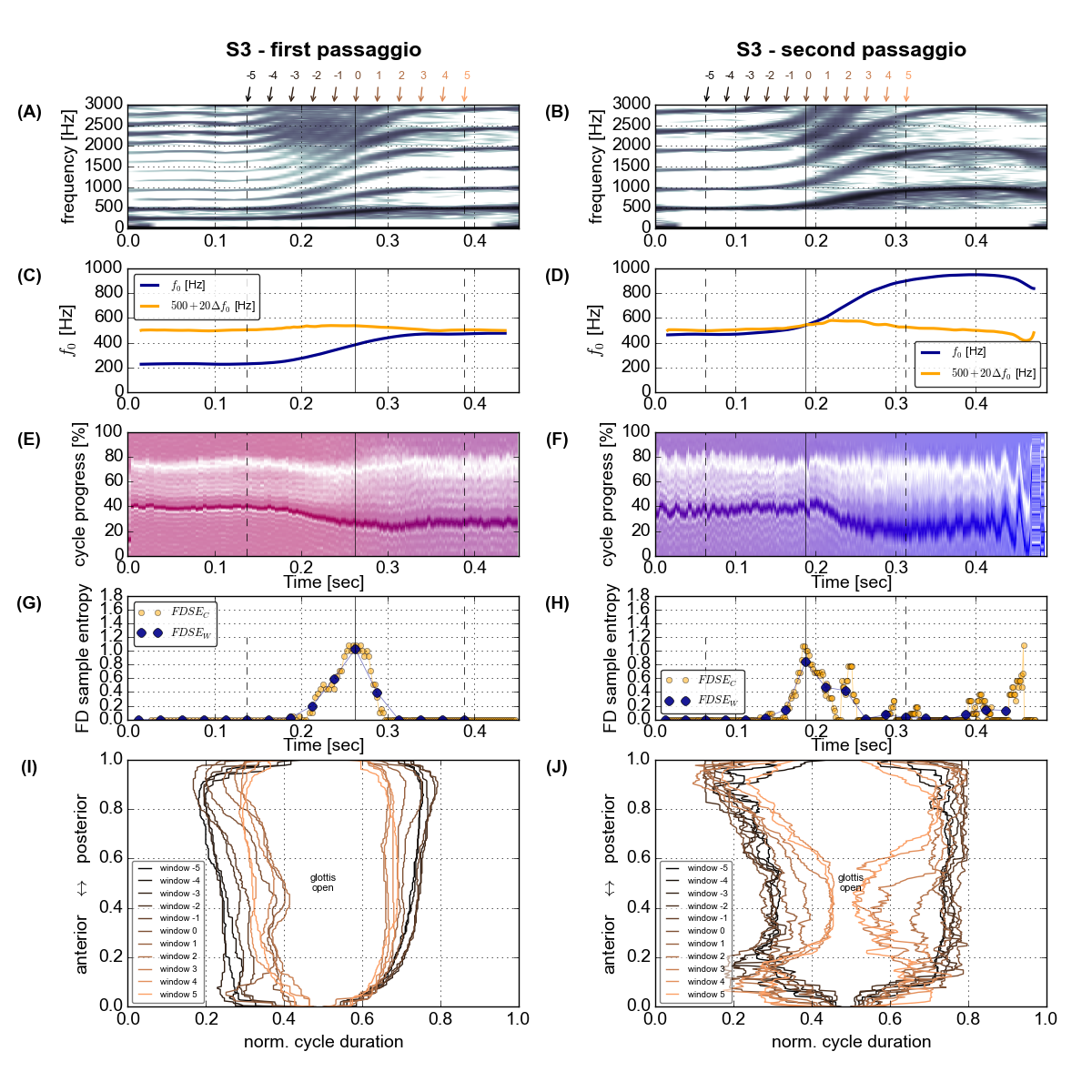

Supplement: S3 Fig — Acoustic spectrogram (window length 1024 frames, 65 dB dynamic range, A and B), time-varying fundamental frequency (ƒo, C and D), dEGG Wavegram (E and F), cycle based (c) and windows based (w) Fourier Descriptors Sample Entropy (FDSE) (G and H), and summary of glottal opening and closure patterns (I and J) for subject 3. (TIF) [file pone.0175865.s005.tif]

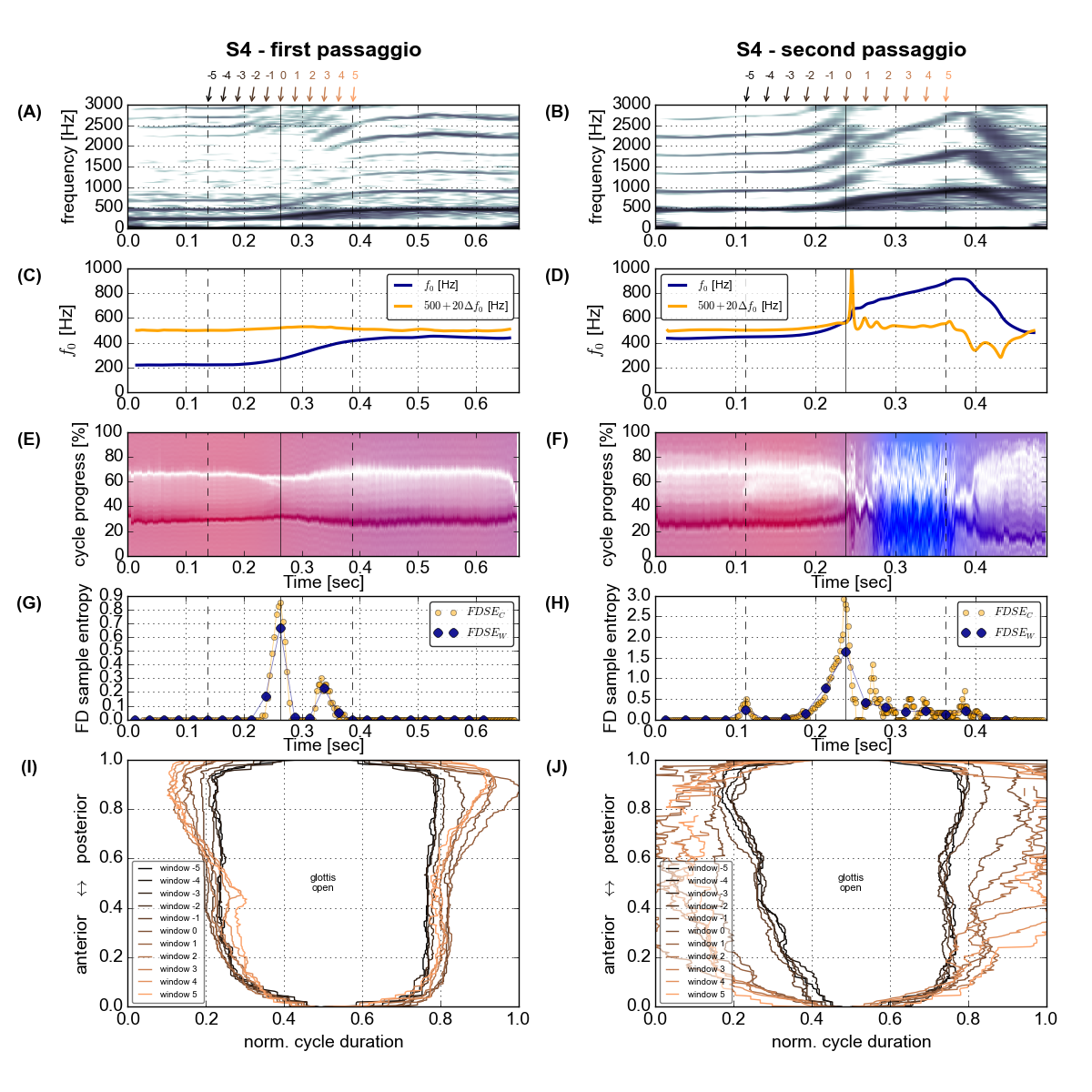

Supplement: S4 Fig — Acoustic spectrogram (window length 1024 frames, 65 dB dynamic range, A and B), time-varying fundamental frequency (ƒo, C and D), dEGG Wavegram (E and F), cycle based (c) and windows based (w) Fourier Descriptors Sample Entropy (FDSE) (G and H), and summary of glottal opening and closure patterns (I and J) for subject 4. (TIF) [file pone.0175865.s006.tif]

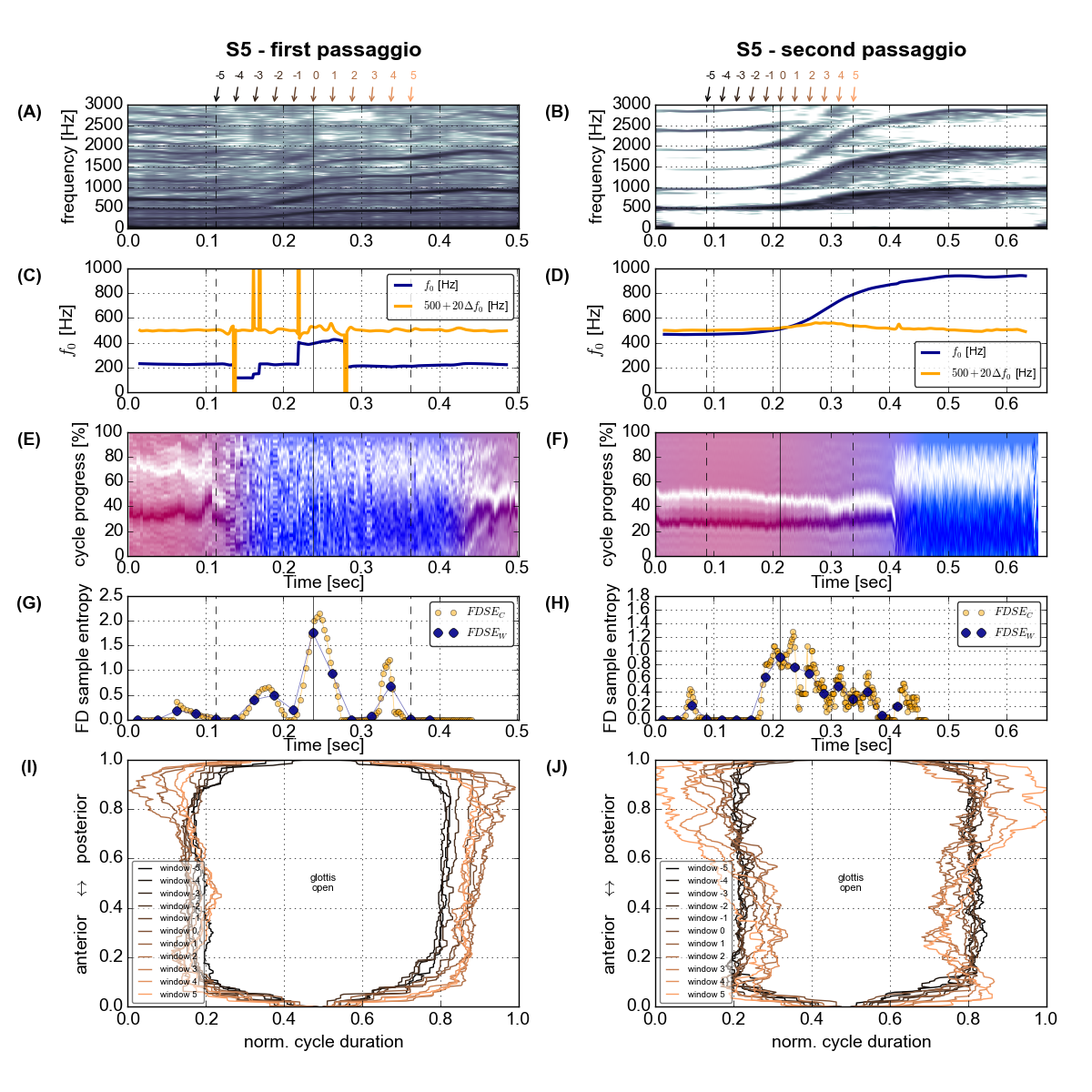

Supplement: S5 Fig — Acoustic spectrogram (window length 1024 frames, 65 dB dynamic range, A and B), time-varying fundamental frequency (ƒo, C and D), dEGG Wavegram (E and F), cycle based (c) and windows based (w) Fourier Descriptors Sample Entropy (FDSE) (G and H), and summary of glottal opening and closure patterns (I and J) for subject 5. (TIF) [file pone.0175865.s007.tif]

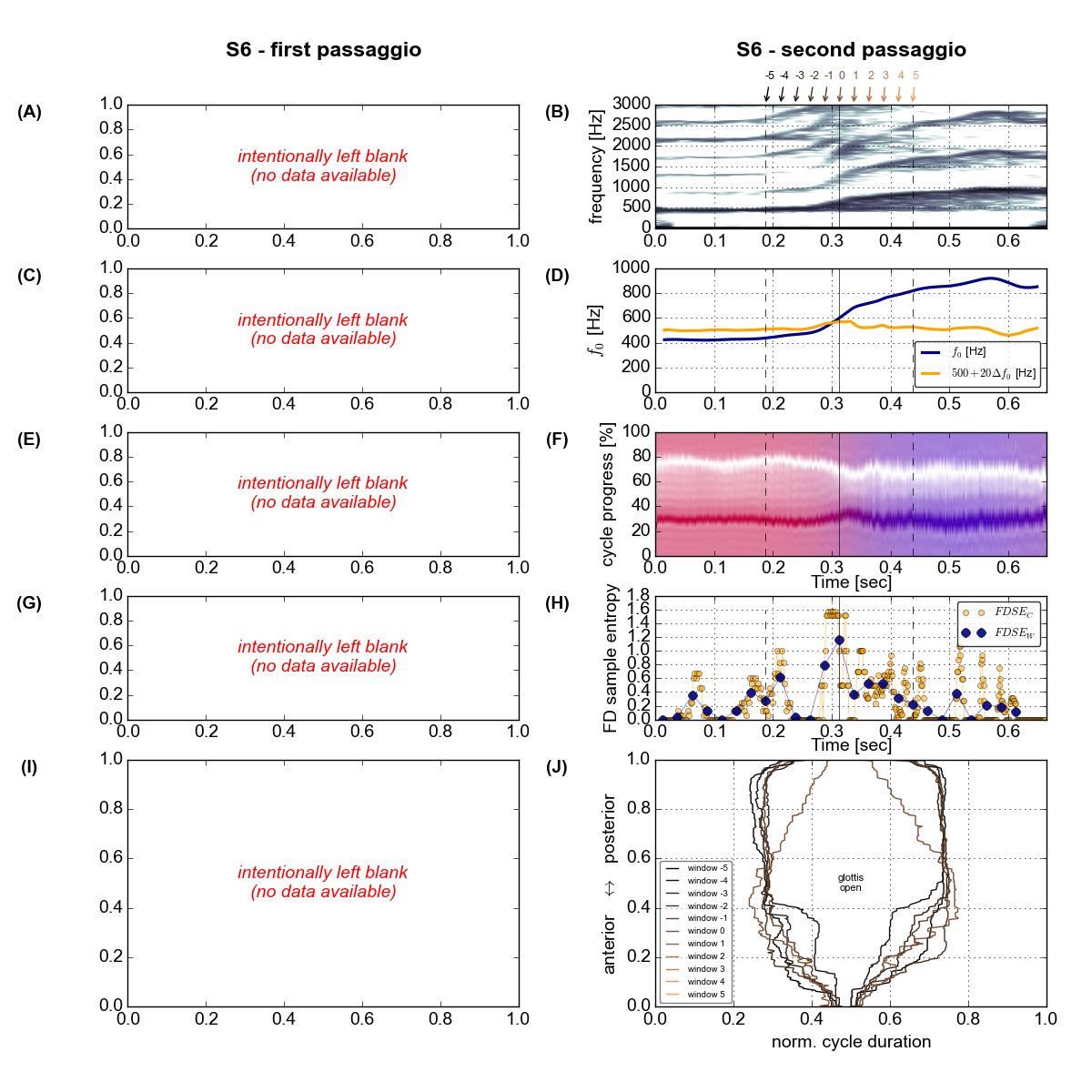

Supplement: S6 Fig — Acoustic spectrogram (window length 1024 frames, 65 dB dynamic range, A and B), time-varying fundamental frequency (ƒo, C and D), dEGG Wavegram (E and F), cycle based (c) and windows based (w) Fourier Descriptors Sample Entropy (FDSE) (G and H), and summary of glottal opening and closure patterns (I and J) for subject 6. (TIF) [file pone.0175865.s008.tif]

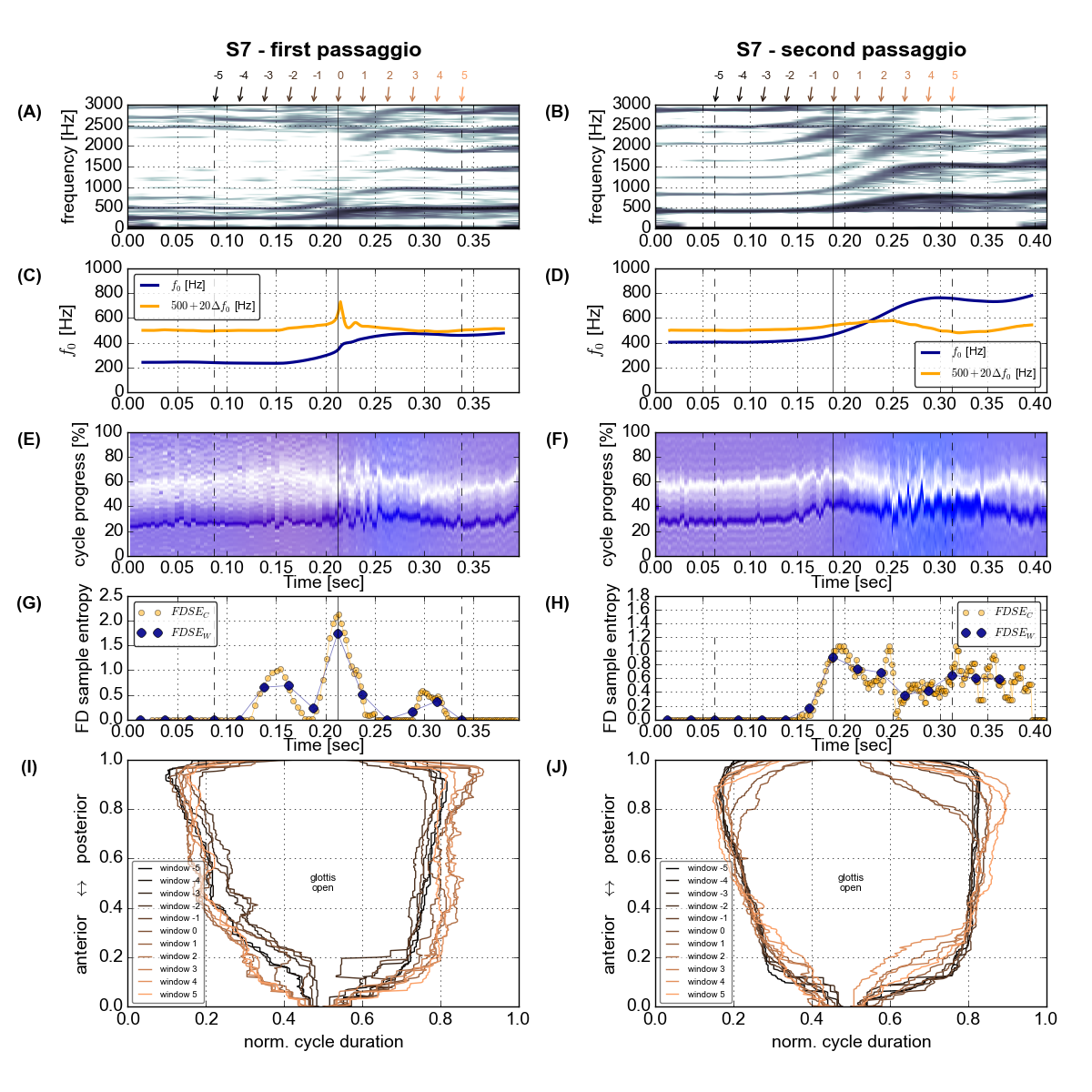

Supplement: S7 Fig — Acoustic spectrogram (window length 1024 frames, 65 dB dynamic range, A and B), time-varying fundamental frequency (ƒo, C and D), dEGG Wavegram (E and F), cycle based (c) and windows based (w) Fourier Descriptors Sample Entropy (FDSE) (G and H), and summary of glottal opening and closure patterns (I and J) for subject 7. (TIF) [file pone.0175865.s009.tif]

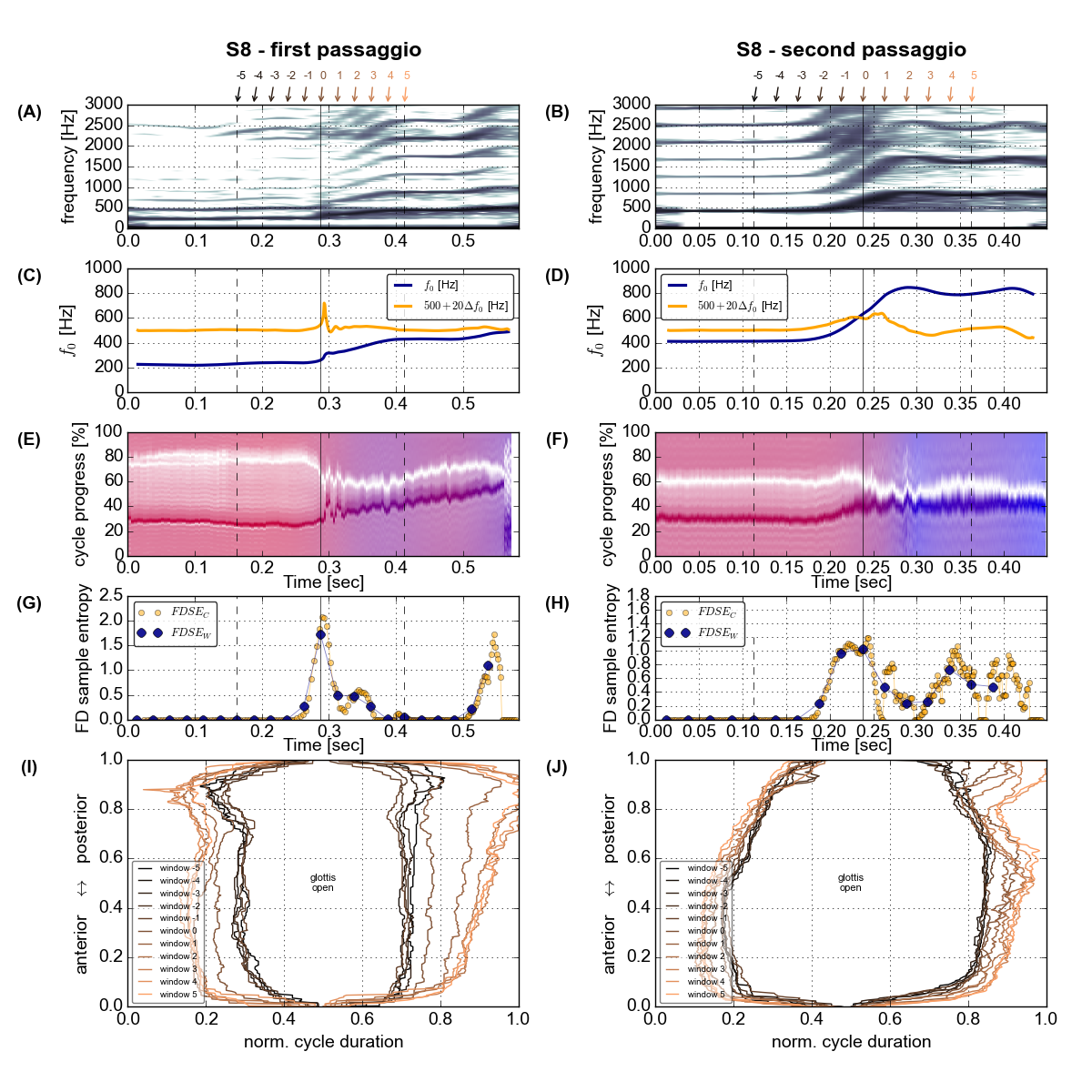

Supplement: S8 Fig — Acoustic spectrogram (window length 1024 frames, 65 dB dynamic range, A and B), time-varying fundamental frequency (ƒo, C and D), dEGG Wavegram (E and F), cycle based (c) and windows based (w) Fourier Descriptors Sample Entropy (FDSE) (G and H), and summary of glottal opening and closure patterns (I and J) for subject 8. (TIF) [file pone.0175865.s010.tif]

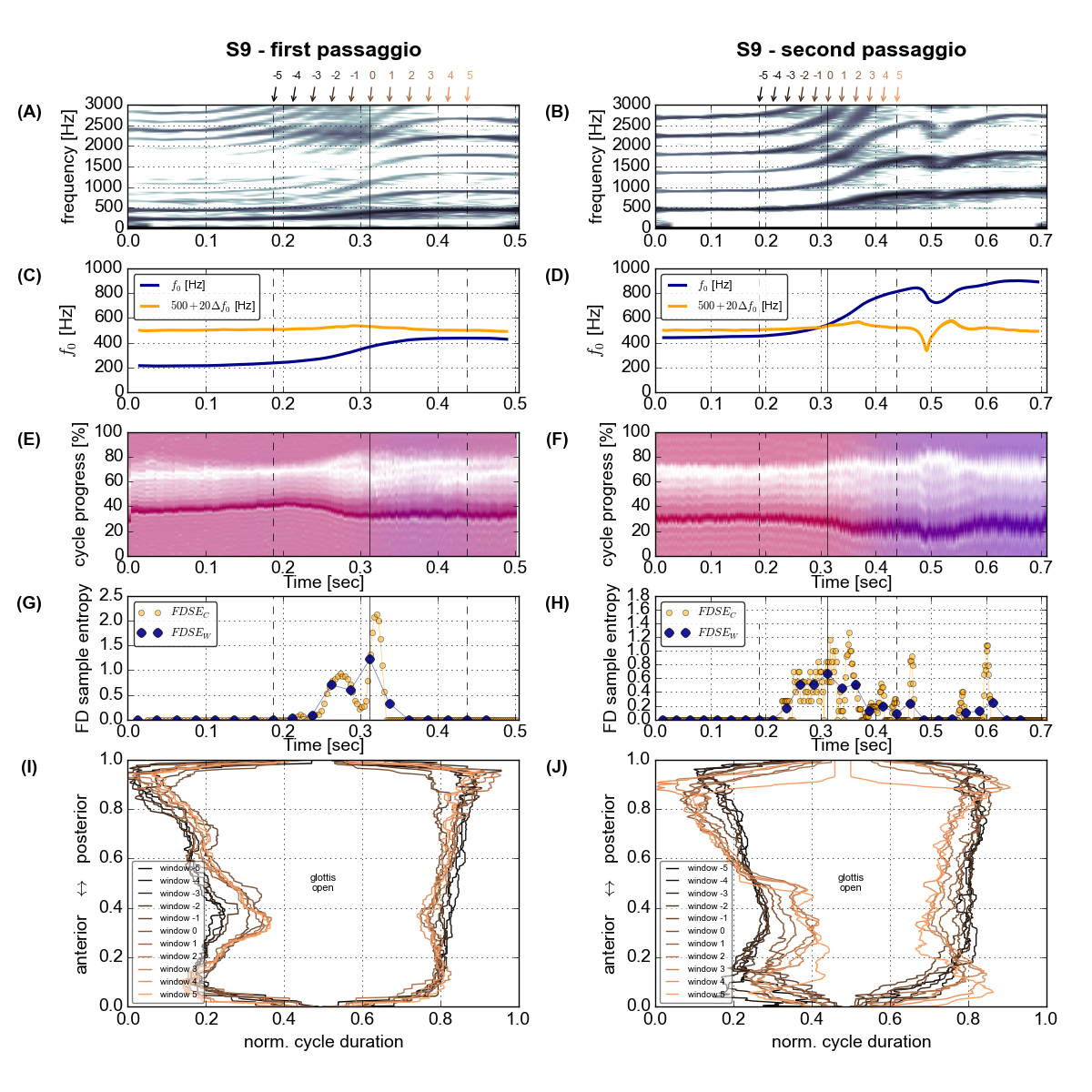

Supplement: S9 Fig — Acoustic spectrogram (window length 1024 frames, 65 dB dynamic range, A and B), time-varying fundamental frequency (ƒo, C and D), dEGG Wavegram (E and F), cycle based (c) and windows based (w) Fourier Descriptors Sample Entropy (FDSE) (G and H), and summary of glottal opening and closure patterns (I and J) for subject 9. (TIF) [file pone.0175865.s011.tif]

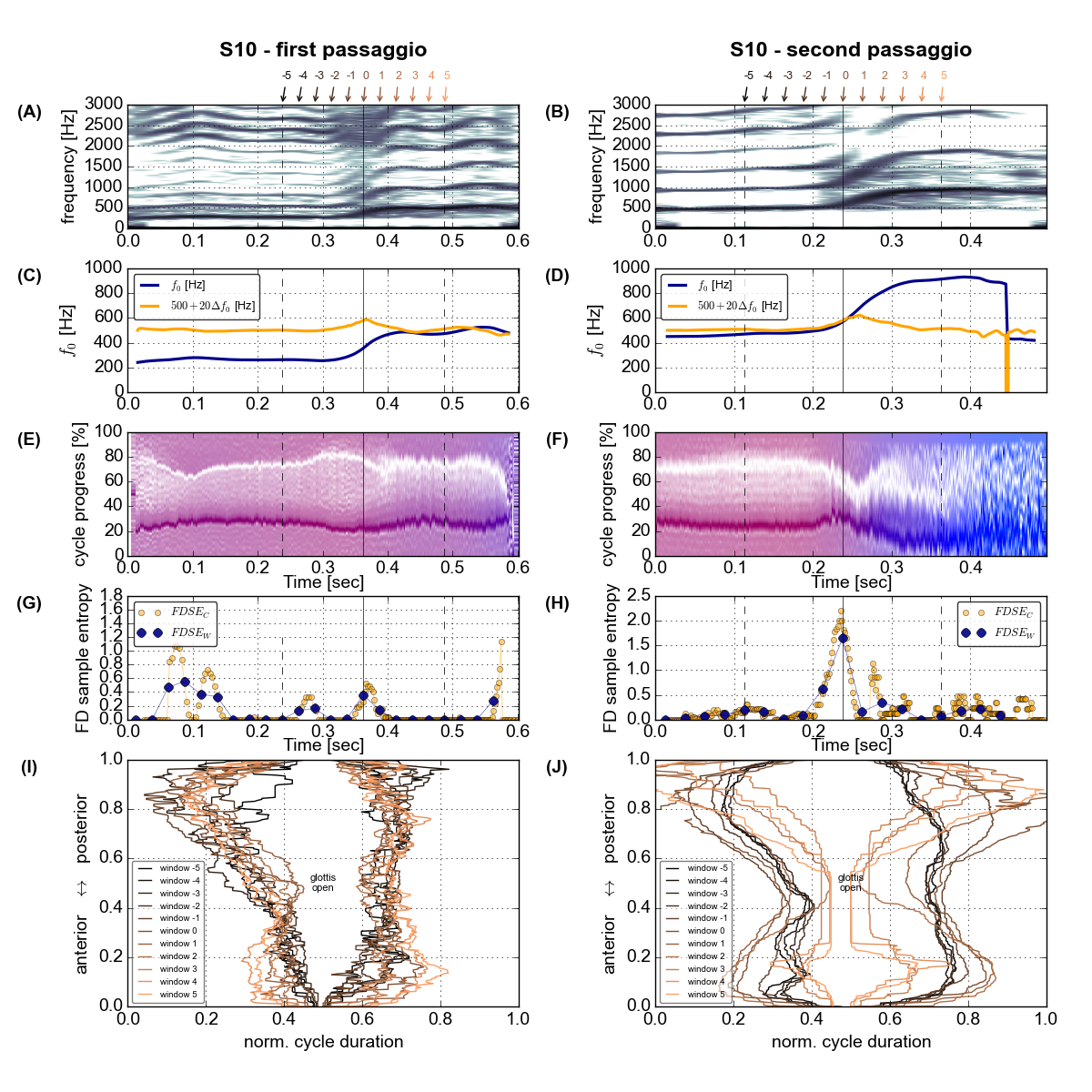

Supplement: S10 Fig — Acoustic spectrogram (window length 1024 frames, 65 dB dynamic range, A and B), time-varying fundamental frequency (ƒo, C and D), dEGG Wavegram (E and F), cycle based (c) and windows based (w) Fourier Descriptors Sample Entropy (FDSE) (G and H), and summary of glottal opening and closure patterns (I and J) for subject 10. (TIF) [file pone.0175865.s012.tif]

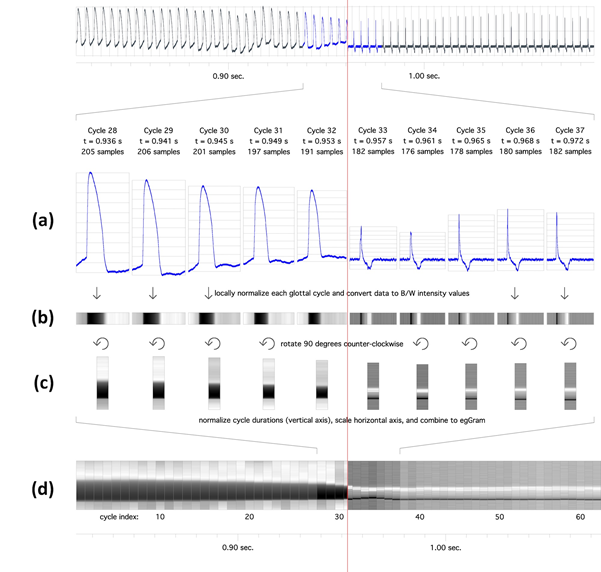

Supplement: S11 Fig — The two halves of the image illustrate the creation of wavegrams based on the EGG (left) and the dEGG signal (right), respectively. (TIF) [file pone.0175865.s013.tif]
